# Chemically Recyclable Polymer System Based on Nucleophilic Aromatic Ring-Opening Polymerization

**DOI:** 10.1021/jacs.3c03455

**Published:** 2023-06-12

**Authors:** Yong-Liang Su, Liang Yue, Huan Tran, Mizhi Xu, Anthony Engler, Rampi Ramprasad, H. Jerry Qi, Will R. Gutekunst

**Affiliations:** † School of Chemistry and Biochemistry, Georgia Institute of Technology, Atlanta, Georgia 30332, United States; ‡ School of Mechanical Engineering, Georgia Institute of Technology, Atlanta, Georgia 30332, United States; § School of Materials Science and Engineering, Georgia Institute of Technology, Atlanta, Georgia 30332, United States; ∥ School of Chemical and Biomolecular Engineering, Georgia Institute of Technology, Atlanta, Georgia 30332, United States

## Abstract

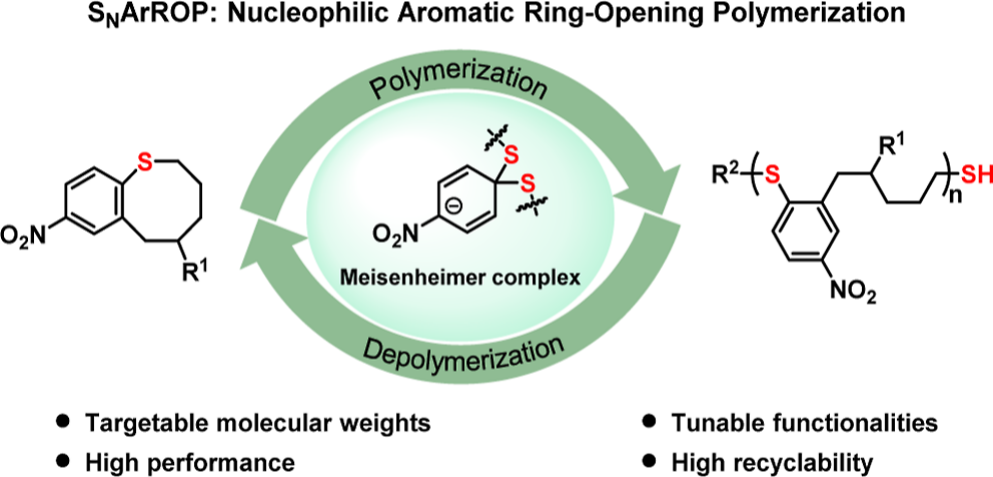

The development of chemically recyclable polymers with desirable properties is a long-standing but challenging goal in polymer science. Central to this challenge is the need for reversible chemical reactions that can equilibrate at rapid rates and provide efficient polymerization and depolymerization cycles. Based on the dynamic chemistry of nucleophilic aromatic substitution (S_N_Ar), we report a chemically recyclable polythioether system derived from readily accessible benzothiocane (**BT**) monomers. This system represents the first example of a well-defined monomer platform capable of chain-growth ring-opening polymerization through an S_N_Ar manifold. The polymerizations reach completion in minutes, and the pendant functionalities are easily customized to tune material properties or render the polymers amenable to further functionalization. The resulting polythioether materials exhibit comparable performance to commercial thermoplastics and can be depolymerized to the original monomers in high yields.

## Introduction

Synthetic polymers are widely used in daily life owing to their wide-ranging functionalities and properties. However, their mass production and consumption, coupled with high durability, result in enormous plastic waste at the end of their useful life.^1−3^ This end-of-life issue of polymers not only has caused serious environmental and economic challenges but also makes them unsustainable if they cannot be recycled, as 90% of synthetic polymers are derived from finite fossil fuels.^4^ Existing approaches to addressing the sustainability issue include mechanical recycling,^5−7^ upcycling to value-added chemicals,^8−12^ and chemical recycling to monomer (CRM),^13−16^ among which CRM enables an attractive circular monomer life cycle and economy.

**Figure 1:**
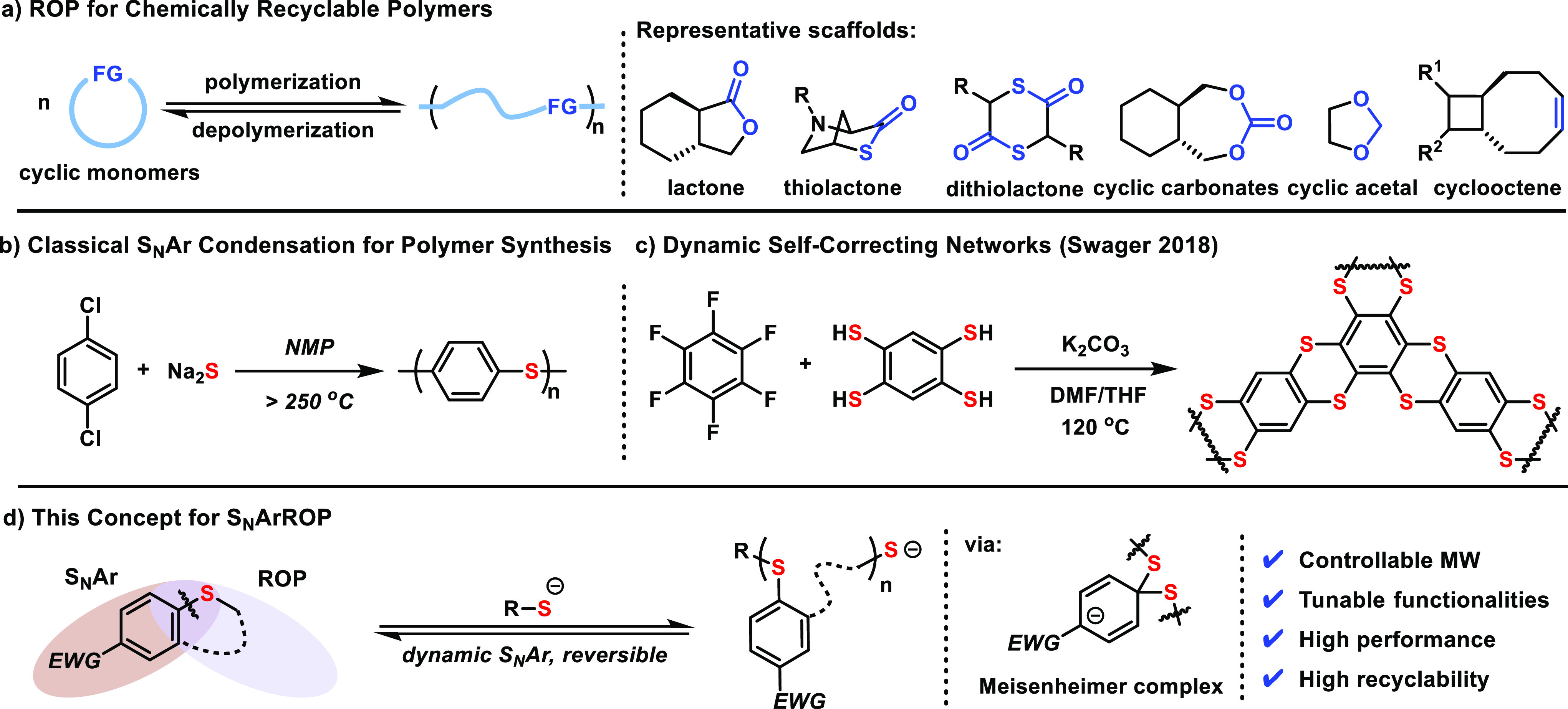
Designs of nucleophilic aromatic ring-opening polymerization (S_N_ArROP). (a) Established cyclic monomer scaffolds for preparing chemically recyclable polymers. (b) Classical S_N_Ar condensation for the preparation of polyphenylene sulfide (PPS). (c) Dynamic self-correcting S_N_Ar condensation for the synthesis of porous polymer networks. (d) New concept for S_N_ArROP for the synthesis of chemically recyclable polythioethers. The combination of dynamic S_N_Ar chemistry and an appropriate ring size for ROP enable the reversibility of this S_N_ArROP strategy.

Several classes of designed polymers that are suitable for CRM, such as polyesters,^17−20^ polythioesters,^21−24^ polyacetals,^25^ polycarbonates,^26^ fused cyclooctene derivatives,^27^ and others,^28−32^ have been recently developed (Figure 1a). A common feature among all of these monomer classes is propagation through a ring-opening polymerization (ROP) mechanism and the presence of modest monomer ring strain that gives rise to a low ceiling temperature (*T*_*c*_). By subjecting these polymers to suitable triggers at elevated temperatures and/or reduced concentrations, the polymers can revert back to their constituent monomers through back-biting and cyclization reactions. Coupled to the overall thermodynamic challenge of identifying monomers with suitable *T*_*c*_ is the challenge of producing polymers with desirable material properties. Chen and co-workers reported the ROP of trans-cyclohexyl-fused γ-butyrolactone, and the polymer exhibited good thermal stability (decomposition temperature *T*_d_ ∼ 340 °C) and high tensile strength (σ_B_ ∼ 55 MPa).^18^ Through a reversible deactivation cationic ROP, Coates and co-workers synthesized poly(1,3-dioxolane), a thermally stable plastic with high tensile strength (σ_B_ ∼ 40 MPa).^25^ Considering the relatively limited application potential of most CRM polymers reported to date, exploration of new concepts for ring-opening polymerization is targeted in this work.

To identify suitable chemistries for a new CRM platform, inspiration was found in the dynamic covalent chemistry of nucleophilic aromatic substitution (S_N_Ar).^33−38^ While numerous high-performance materials have been prepared and industrially used through S_N_Ar polymerization (Figure 1b), limited reports have taken advantage of the chemistry’s inherent reversibility.^39−41^ The pioneering work by Swager and Ong leveraged this feature to design error-correcting aryl sulfide network polymers (Figure 1c).^42^ To merge this concept with the potential for recyclability to monomer, a cyclic aromatic thioether capable of S_N_Ar was envisioned that could be polymerized through nucleophilic aromatic ring-opening polymerization (S_N_ArROP) (Figure 1d). While some macrocyclic systems have been shown to polymerize, a well-defined monomer for chain-growth ring-opening polymerization through S_N_Ar chemistry has yet to be realized.^43^

## Results and Discussion

### Monomer Design and Synthesis

**Figure 2:**
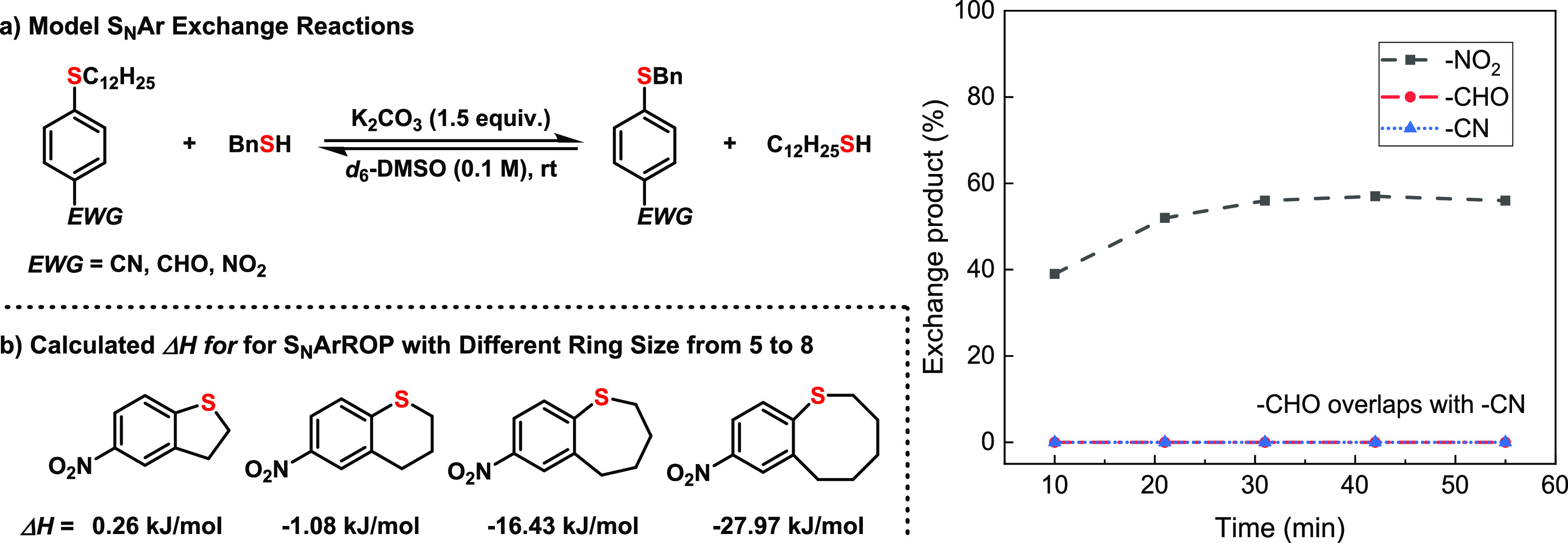
Identifying the appropriate electron withdrawing group and ring size for the **BT** monomers. (a) Exchange reactions showing rapid equilibrium established with *p*-NO_2_ substituted phenyl sulfide substrate at room temperature but no conversion from the −CN, and −CHO substrates. (b) Calculated Δ*H* for S_N_ArROP with different ring sizes from 5 to 8, suggesting enthalpically favorable polymerization of 7- and 8-membered ring substrates.

**Figure 3:**
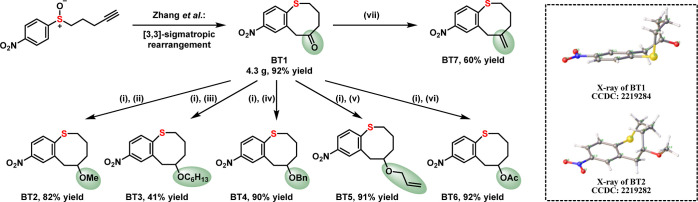
Synthesis and characterization of the **BT** monomers. Synthesis of the 8-membered ring substrate **BT1** through an efficient [3,3]-sigmatropic rearrangement and its convenient transformation to **BT2**-**BT7**. (i) NaBH_4_, MeOH, 0 °C. (ii) NaH, MeI, THF. (iii) NaH, C_6_H_13_I, THF. (iv) NaH, BnBr, DMF. (v) NaH, allyl bromide, DMF. (vi) Ac_2_O, DMAP, pyridine. (vii) ^*t*^BuOK, PPh_3_PMeBr, THF. The X-ray of **BT1** and **BT2** are shown as the insert.

To identify the feasibility of a ring-opening monomer for S_N_Ar, exchange reactions were examined between benzyl mercaptan (BnSH) and aryl dodecyl sulfides with different electron-withdrawing para-substituents (CN, CHO, and NO_2_). While *p*-CN and *p*-CHO-substituted substrates provide no conversion at room temperature and low conversion at 60 °C, *p*-NO_2_-phenyl dodecyl sulfide reacted with BnSH smoothly and reached equilibrium in approximately 30 min at room temperature (Figure 2a and Supplementary Figures S7–S9). Based on our recently developed first-principles computational methodology, the ring-opening polymerization (ROP) enthalpy (Δ*H*) for NO_2_-substituted cyclic monomer scaffolds was calculated (Figure 2b).^44^ The results showed that the 7- and 8-membered cyclic aryl thioethers have sufficient Δ*H* values to promote polymerization (−16.43 and −27.97 kJ/mol, respectively), whereas the 5- and 6-membered substrates were predicted to be insufficiently strained (0.26 and −1.08 kJ/mol). Fortunately, the [3,3]-sigmatropic rearrangement of alkynyl sulfoxides reported by Zhang and co-workers offered expedient and scalable access to 8-membered ring aryl sulfides (**BT1**) bearing the requisite nitro-group substitution (Figure 3).^45^ Additionally, the parent ketone (**BT1**) was readily reduced to an alcohol that could be further derivatized with a variety of electrophiles to install side-chain functionalities (**BT2**-**BT6**) or transformed to **BT7** containing an exocyclic C=C double bond through the Wittig reaction (Figure 3). In addition to full characterization of all monomers through ^1^H NMR, ^13^C NMR, and mass spectrometry, X-ray structures of **BT1** and **BT2** were also obtained (Figure 3). The X-ray studies showed that **BT1** and **BT2** exist in the crystal state exclusively in the boat-chair conformation, and there were two rotamers in **BT1** with populations of 97.7 and 2.3%, respectively.

### Polymerization Studies

**Figure 4:**
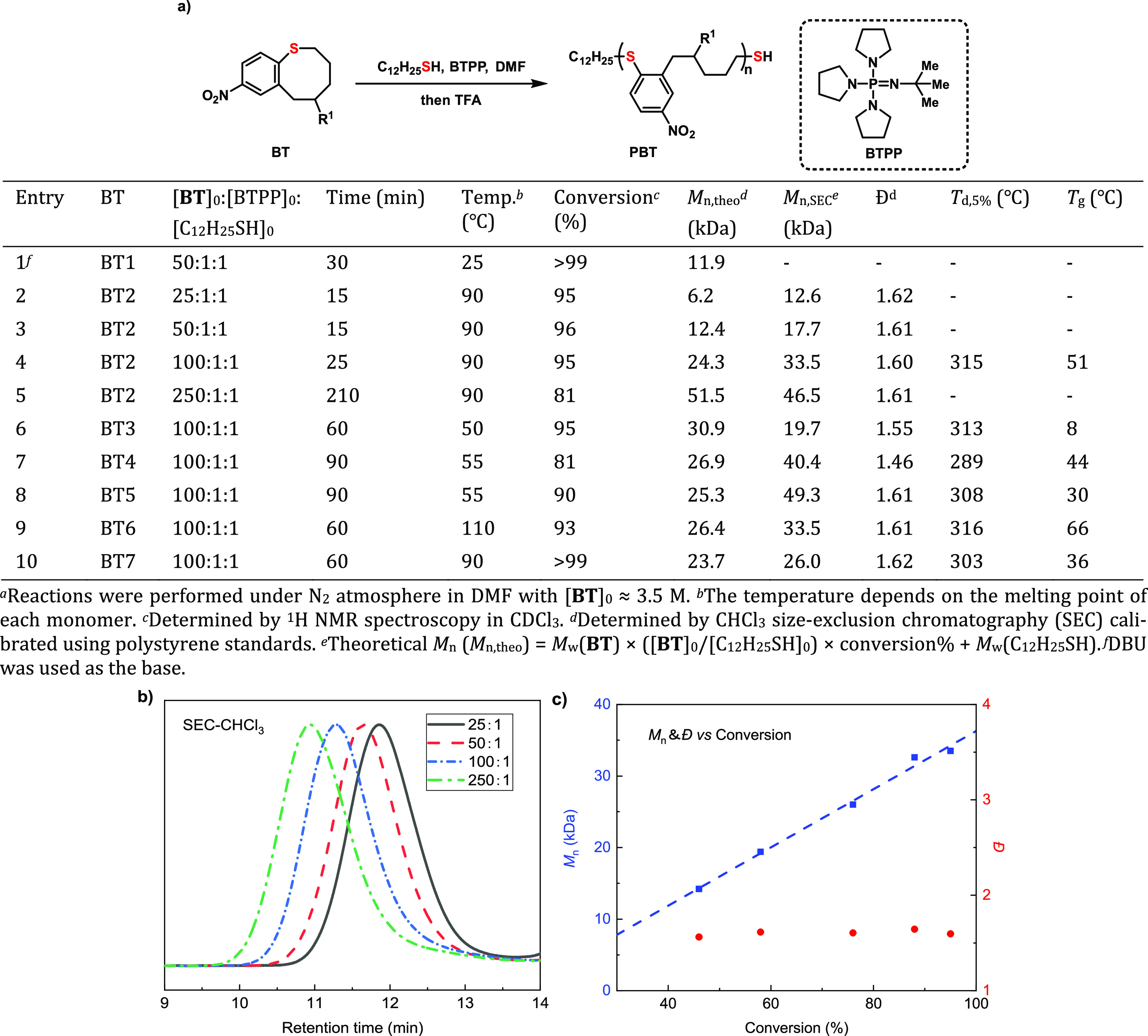
S_N_ArROP of **BT** monomersand characterization of **PBTs**. (a) Results of **BT** polymerizations by BTPP-based catalytic systems. (b) SEC curves for **PBT2** produced at different [**BT2**]_0_/[BTPP]_0_/[C_12_H_25_SH]_0_ ratios. (c) *M*_n_–conversion correlation (blue) and *D̵*–conversion correlation (red) of S_N_ArROP of **BT2**.

At the outset, the polymerizability of **BT1** was probed via measuring its exchange reaction with BnSH, and a rapid exchange reaction was finished between **BT1** and BnSH in DMSO-*d*_6_ with K_2_CO_3_ as the base at room temperature (Supplementary Figure S10). To keep the polymerization homogeneous, the organic base DBU instead of the inorganic base K_2_CO_3_ was used. A preliminary polymerization test with a [**BT1**]_0_/[DBU]_0_/[C_12_H_25_SH]_0_ ratio of 50:1:1 yielded a poorly soluble polymer **PBT1** with almost full conversion (Figure 4a, entry 1). The methyl ether side-chain of **BT2** improved the solubility of the corresponding polymer and facilitated the study of the molecular weight and dispersity through gel permeation chromatography (GPC) while still maintaining high conversions. Solvent choice proved to be critical for polymerization. Fast reaction rates and high conversions were generally found in polar aprotic solvents, including dimethyl sulfoxide (DMSO), dimethylformamide (DMF), dimethylacetamide (DMA), *N*-methyl-2-pyrrolidone (NMP), and *N*,*N*′-dimethylpropyleneurea (DMPU), while no reaction at all in relatively less polar solvents such as chloroform (CHCl_3_), 1,2-dichloroethane (DCE), and tetrahydrofuran (THF) (Supplementary Table S1). These results can be explained by the requirement of a polar environment to stabilize the Meisenheimer intermediate (Figure 1d) in the nucleophilic aromatic substitution step.^46−48^ While the experimental *M*_n_ values were slightly higher than those calculated from the ratio of [*M*]_0_/[*I*]_0_ and conversion, targetable molecular weights were readily obtained. A small peak in the lower molecular weight range was observed in the GPC traces, suggesting the possible formation of cyclic oligomer byproducts, which was further confirmed by matrix-assisted laser desorption/ionization-time-of-flight (MALDI-TOF) mass spectrometry (Supplementary Figures S12 and S13). By increasing the monomer concentration, the cyclic oligomer byproducts were limited, and a unimodal high-molecular-weight polymers were obtained (Supplementary Table S2). Interestingly, the polymerization was found to readily proceed to high conversion at a wide range of temperatures (90 to 140 °C) (Supplementary Table S2). A variety of bases and thiol initiators were further screened, and strong bases with higher *p*K_a_ and more nucleophilic thiolates were found beneficial for the polymerization (Supplementary Tables S4 and S5).

Under the optimized reaction conditions, a series of molecular weight targets were obtained by adjusting the monomer-to-initiator ratio ([**BT2**]_0_/[BTPP]_0_/[C_12_H_25_SH]_0_) from 25:1:1 to 250:1:1 (Figure 4a, entries 2–5). Moreover, the molecular weight increased linearly as the monomer to initiator ratio increased, while the corresponding dispersity (*Đ*) remained at a modest value (∼1.60) during polymerization (Figure 4b). These data strongly support a chain-growth mechanism to the polymerization with chain-transfer events occurring between propagating polymers and the aryl sulfide backbone. Next, the generality of the S_N_ArROP strategy was investigated using monomers bearing different substituents (O–C_6_H_13_, O–Bn, O–allyl, O–Ac, and C=C). The S_N_ArROP of these monomers was achieved at a temperature slightly higher than their melting points at a 3.5 M monomer concentration to ensure solubility throughout the polymerization (Figure 4a, entries 6–10). The corresponding polymers with molecular weight ranging from 19.7 to 49.3 kDa and consistent *Đ* values ranging from 1.46 to 1.62 were obtained from a [**BT**]_0_/[BTPP]_0_/[C_12_H_25_SH]_0_ ratio of 100:1:1. The substituents showed a slight influence on the polymerization reactivity, as shown by the conversion ranging from 81 to 99%. The convenient introduction of these various functional groups enables the tuning of the resulting material’s properties as shown below.

### Thermal and Mechanical Properties

Thermogravimetric analysis (TGA) and differential scanning calorimetry (DSC) were used to measure the thermal properties for each poly(aryl thioether). All the produced polymers obtained with [**BT**]_0_/[BTPP]_0_/[C_12_H_25_SH]_0_ = 100:1:1 displayed high thermal stability with a range of thermal decomposition temperature *T*_d_ (defined as the temperature causing a 5% weight loss) from 289 to 316 °C (Figure 4a and Supplementary Figure S16). Depending on the pendant groups on the monomers, a wide range of *T*_g_s from 8 to 66 °C can be accessed (Figure 4a and Supplementary Figure S16). The absence of clear melting transitions indicates the amorphous character of these polymers, which could be explained by atactic microstructures generated from the racemic monomers.

**Figure 5:**
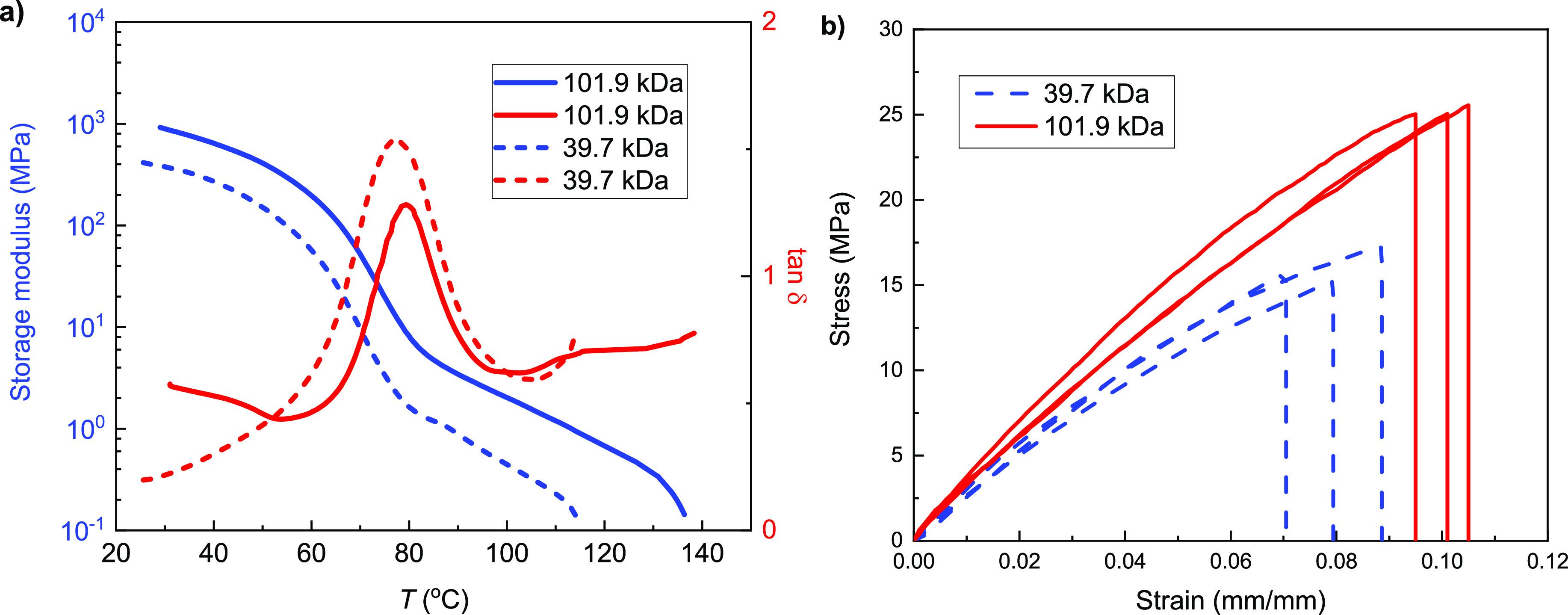
Mechanical properties of **PBT2**. (a) DMA storage modulus and tan δ profiles of **PBT2** with different molecular weights. (b) Tensile stress–strain curves of **PBT2** with different molecular weights.

Further investigation was focused on the thermal and mechanical properties of these PBT samples, especially the effect of the molecular weight on these properties. **PBT2** with two different *M*_n_ (39.7 kDa and 101.9 kDa) was prepared. **PBT2**s exhibited the typical behavior of a thermoplastic polymer as shown by dynamic mechanical analysis (DMA) (Figure 5). Both samples possessed relatively high storage modulus (*E*′) at room temperature as glassy polymers. However, after the glass transition region with *T*_g_ values around 80 °C (as defined by the peak maxima of tanδ), *E′* continually decreased until a quick drop to the viscous flow state due to melting (Figure 5a). The sample with a higher molecular weight displayed both higher *E′* and *T*_g_ as expected (*M*_n_ = 39.7 kDa: *E′* = 375.1 MPa (30 °C), *T*_g_ = 77.8 °C; *M*_n_ = 101.9 kDa: *E′* = 1243.8 MPa (30 °C), *T*_g_ = 79.5 °C). Furthermore, tensile testing of the **PBT2** showed the same trend as a glassy polymer with Young̀s modulus *E* = 275.6 ± 19.9 and 349.9 ± 18.76 MPa, ultimate tensile strength σ = 16.07 ± 0.84 and 25.21 ± 0.24 MPa, and elongation at break ε = 7.95 ± 0.74 and 10.03 ± 0.41% for the 39.7 and 101.9 kDa samples, respectively (Figure 5b). The tensile performance is within the range of some commonly used thermoplastics such as polyethylene (PE), ethylene vinyl acetate (EVA), and polyvinyl chloride (PVC), suggesting applications potential upon further increases to molecular weight or the addition of plasticizers.

### Chemical Recyclability

**Figure 6:**
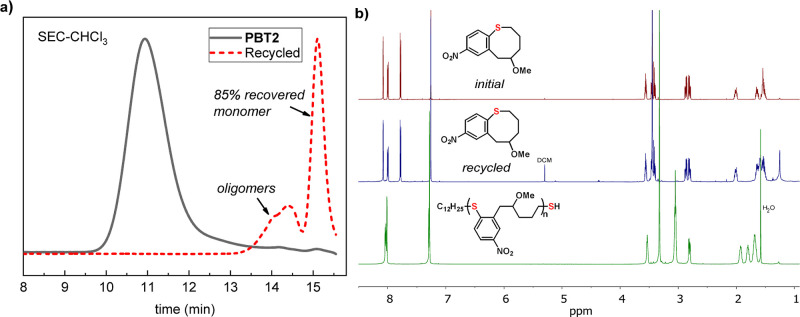
Chemical recyclability of **PBT2**. (a) SEC curves for depolymerization of **PBT2** (46.5 kDa) with substoichiometric DBU and C_12_H_25_SH (0.55 equiv relative to repeat units). (b) Overlays of ^1^H NMR spectra of initial and recycled **BT2** and **PBT2**.

As an effort to achieve the original purpose of designing recyclable polymers with robust and durable behavior, the advantages of PBTs in mechanical properties led us to explore the recyclability to monomers of these polymers. The initial attempts to depolymerize the parent polymer **PBT2** from the chain end by adding the DBU were unsuccessful, but the introduction of additional thiol was found to help conversion through cleaving segments from the polymer backbone for depropagation (Supplementary Figure S19 and Figure S20). By adding 0.55 equivalent of DBU and C_12_H_25_SH relative to the repeat unit at a temperature of 90 °C and a concentration of 10 mg/mL, an isolated yield of 85% for **BT2** was achieved (Figure 6). This depolymerization is likely initiated through backbone cleavage due to reversible S_N_Ar chemistry, which is further supported by the successful depolymerization of an end-capped **PBT5** (capped with iodoacetamide) producing monomer **BT5** in 70% yield (Supplementary Figure S21).

## Conclusions

In conclusion, the dynamic reversibility of S_N_Ar chemistry and ring-opening polymerization have been successfully merged into a well-defined monomer platform for the first time. S_N_ArROP of **BT**s is an effective and powerful strategy for the synthesis of chemically recyclable polythioethers. The facile modification of the monomer scaffold leads to readily tunable material properties, and the high recyclability suggests that the **BT** platform is a robust candidate for the design of new dynamic and depolymerizable polymers capable of CRM. Further studies on post-polymerization modification to chemically crosslink and 3D print recyclable materials are currently underway.

## Supporting Information Available

Experimental procedure and spectroscopic data for all new compounds and X-ray crystallographic data for BT1 and BT2 (PDF).
