# Supplementary material for: Chemically Recyclable Polymer System Based on Nucleophilic Aromatic Ring-Opening Polymerization
Source: J Am Chem Soc. 2023 Jun 12;145(25):13950–6. doi: 10.1021/jacs.3c03455 (PMC10311534; doi:10.1021/jacs.3c03455)
Supplement: Supplementary file 1 — ja3c03455_si_001.pdf [file ja3c03455_si_001.pdf]

Supporting Information

A chemically recyclable polymer system based on nucleophilic aromatic ring-opening polymerization

Yong-Liang Su[1], Liang Yue[2], Huan Tran[3], Mizhi Xu[1], Anthony Engler[4], Rampi Ramprasad[3], H. Jerry Qi[2] and Will R. Gutekunst[1]*

[1]*School of Chemistry and Biochemistry, Georgia Institute of Technology, 901 Atlantic Drive NW, Atlanta, Georgia 30332, United States.*

[2]*School of Mechanical Engineering, Georgia Institute of Technology, Atlanta, Georgia 30332, United States*

[3]*School of Materials Science and Engineering, Georgia Institute of Technology, Atlanta, Georgia 30332, United States*

[4]*School of Chemical and Biomolecular Engineering, Georgia Institute of Technology, Atlanta, Georgia 30332, United States.*

* E-mail: *willgute@gatech.edu*

Table of Contents

1. General information S2
2. Synthesis of monomers S2
3. Optimization of the polymerization conditions S6
4. General polymerization procedure and characterization of polymers S8
5. Model S_NAr exchange reaction S11
6. Analysis of cyclic oligomer by-products and the linear structure of PBT S15
7. TGA and DSC Studies of the PBTs S17
8. Mechanical properties of the PBT2s S17
9. Chemical recycle of PBTs S18
10. Polymerization thermodynamic studies of BT2 .... S21
11. X-ray crystal structure of the monomers BT1 and BT2 .... S22
12. Calculations of ring-opening enthalpy ΔH S25
13. NMR spectrum of products S27
14. References S41

1. General information

General methods. All reactions were carried out under a nitrogen atmosphere with dry solvents using anhydrous conditions unless otherwise stated. Dry, degassed *N*,*N*-dimethylformamide (DMF), acetonitrile (CH_3CN), and tetrahydrofuran (THF) were obtained from a JC Meyer solvent purification system. Dimethyl sulfoxide (DMSO), dimethylacetamide (DMA), *N*-methyl-2-pyrrolidone (NMP), *N*,*N*′-dimethylpropyleneurea (DMPU), nitrobenzene ($PhNO_2$), chloroform ($CHCl_3$) and 1,2-dichloroethane (DCE) were purchased from commercial sources and further dried with activated 4Å molecular sieves and degassed. Unless otherwise stated, all other reagents were purchased at the highest commercial quality and used without further purification. Yields refer to chromatographically and spectroscopically (^{1}H-NMR) homogeneous materials, unless otherwise stated. Reactions were monitored by thin layer chromatography (TLC) carried out on 0.25 mm E. Merck silica gel plates (60F-254) using UV light as the visualizing agent and basic aqueous potassium permanganate ($KMnO_4$), and heat as developing agents. E. Merck silica gel (60, particle size 0.043–0.063 mm) was used for flash column chromatography. NMR spectra were recorded on Bruker Avance 400, 500 or 700 MHz instruments and calibrated using residual undeuterated solvent as an internal reference ($CHCl_3$ @ 7.26 ppm ^{1}H NMR, 77.16 ppm ^{13}C NMR). The following abbreviations (or combinations thereof) were used to explain the multiplicities: s = singlet, d = doublet, t = triplet, q = quartet, m = multiplet, br = broad, comp = composite of magnetically non-equivalent protons. Mass spectra (MS) were recorded on LC/MS (Agilent Technologies 1260 Infinity II/6120 Quadrupole) or a time-of-flight matrix assisted laser desorption/ionization (MALDI-TOF) using a *trans*-2-[3-(4-*tert*-butylphenyl)-2-methyl-2-propenylidene]malononitrile (DCTB) matrix. Polymer samples were analyzed using a Tosoh EcoSEC HLC 8320GPC system with TSKgel SuperHZ-L columns eluting $CHCl_3$ containing 0.25% NEt_3 at a flow rate of 0.45 mL/min. All number-average molecular weights and dispersities were calculated from refractive index chromatograms using PStQuick Mp-M polystyrene standards. Thermogravimetric analyses (TGA) were performed under nitrogen atmosphere on a Pyris 1 TGA (PerkinElmer) at a heating rate of 10 °C/min. Differential scanning calorimetry (DSC) analyses were measured on a DSC 3+ STARe system (Mettler Toledo). The reported data were obtained from the third heating cycle at a heating rate of 10 °C/min. Melting points were measured on a MEL-TEMP II Laboratory Devices (uncorrected).

2. Synthesis of monomers

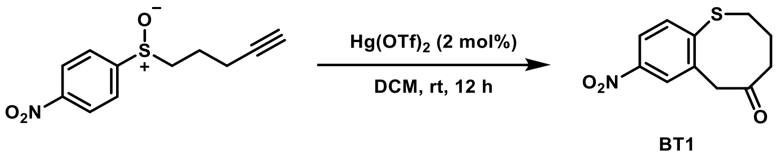

The monomer **BT1** was prepared according to the reported literature.[1] To an oven-dried flask was added sulfoxide (4.75 g, 20.0 mmol), $Hg(OTf)_2$ (199.5 mg, 2 mol %). and dry DCM (400 mL). The mixture was stirred for 12 h at the room temperature. The reaction mixture was concentrated under vacuum. The residue was purified by column chromatography on silica gel (eluent: hexanes/ethyl acetate = 3:1) to give product **BT1** (yellow solid, 92% yield, 4.37 g).

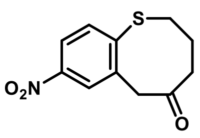

BT1

8-Nitro-3,4-dihydro-2*H*-benzo[*b*]thiocin-5(6*H*)-one.
^{1}H NMR (700 MHz, chloroform-*d*) δ 8.22 – 8.09 (comp, 2H), 7.95 – 7.84 (m, 1H), 3.99 (s, 2H), 2.93 – 2.89 (m, 2H), 2.33 – 2.28 (m, 2H), 2.21 – 2.16 (m, 2H). **^{13}C NMR** (176 MHz, chloroform-*d*) δ 209.3, 148.6, 143.9, 143.7, 138.9, 124.8, 123.5, 51.4, 39.7, 39.2, 30.6. **MS (m/z)**: calcd for $C_{11}H_{12}NO_3S$, $[M+H]^+$: 238.05; found, 238.2. The spectral data were in accordance with those reported in the literature.[2] **Melting point**: 133-135 °C.

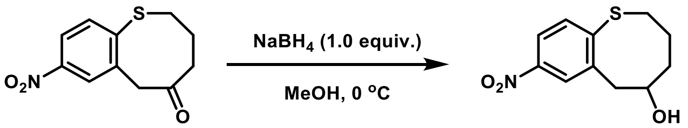

To a flask were added **BT1** (3.09 g, 13.0 mmol), MeOH (50 mL) and a stirring bar, and the system was cooled to 0 °C. Then $NaBH_4$ (491.8 mg, 13.0 mmol, 1.0 equiv.) was added in portions and the mixture was stirred for 30 min. The reaction was quenched with H_2O (30 mL) and the mixture was extracted with DCM (3×50 mL). The combined organic layers were washed with brine, dried over anhydrous Na_2SO_4 and concentrated *in vacuo*. The crude mixture was purified by column chromatography on silica gel (eluent: hexanes/ethyl acetate = 2:1) to give product **BTS1** (white solid, quantitative yield, 3.08 g).

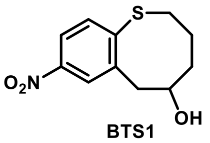

BTS1

8-Nitro-3,4,5,6-tetrahydro-2*H*-benzo[*b*]thiocin-5-ol
^{1}H NMR (700 MHz, chloroform-*d*) δ 8.16 (d, *J* = 2.6 Hz, 1H), 8.02 (dd, *J* = 8.4, 2.6 Hz, 1H), 7.80 (d, *J* = 8.4 Hz, 1H), 4.26 – 4.05 (m, 1H), 3.51 (dd, *J* = 13.1, 3.9 Hz, 1H), 3.37 (dd, *J* = 13.1, 7.3 Hz, 1H), 2.91 – 2.73 (m, 2H), 2.12 – 1.96 (m, 1H), 1.69 – 1.58 (m, 2H), 1.50 – 1.36 (m, 1H). **^{13}C NMR** (176 MHz, chloroform-*d*) δ δ 148.1, 145.0, 143.3, 137.2, 125.3, 122.0, 72.4, 40.9, 38.5, 33.7, 24.4. **MS (m/z)**: calcd for $C_{11}H_{14}NO_3S$, $[M+H]^+$: 240.07; found, 240.1. **Melting point**: 100-102 °C.

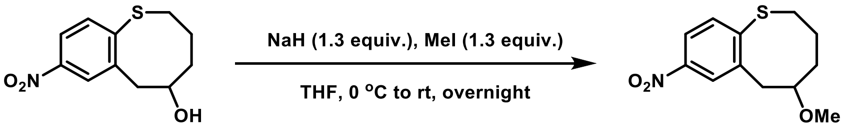

BT2

To a solution of the alcohol substrate (2.39 g, 10.0 mmol) in anhydrous THF (80 mL) were added NaH (0.52 g, 13 mmol, 1.3 equiv.) and MeI (1.84 g, 13 mmol, 1.3 equiv.) under 0 °C. After stirring at rt for overnight, the reaction mixture was quenched by H_2O (30 mL), then extracted with EtOAc (3×60 mL). The combined organic layers were washed with brine, dried over anhydrous Na_2SO_4 and concentrated *in vacuo*. The crude mixture was purified by column chromatography on silica gel (eluent: hexanes/ethyl acetate = 12:1) to give product **BT2** (white solid, 82% yield, 2.08 g).

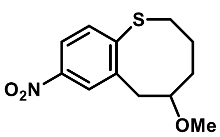

BT2

5-Methoxy-8-nitro-3,4,5,6-tetrahydro-2*H*-benzo[*b*]thiocine
^{1}H NMR (700 MHz, chloroform-*d*) δ 8.08 (d, *J* = 2.5 Hz, 1H), 8.00 (dd, *J* = 8.4, 2.5 Hz, 1H), 7.78 (d, *J* = 8.4 Hz, 1H), 3.56 (tt, *J* = 7.4, 3.9 Hz, 1H), 3.47 – 3.44 (comp, 4H), 3.41 (dd, *J* = 13.1, 7.4 Hz, 1H), 2.87 (ddd, *J* = 14.1, 8.3, 2.9 Hz, 1H), 2.80 (ddd, *J* = 14.1, 8.3, 2.9 Hz, 1H), 2.04 – 1.98 (m, 1H), 1.68 – 1.61 (m, 1H), 1.57 – 1.47 (m, 2H). **^{13}C NMR** (176 MHz, chloroform-*d*) δ 148.1, 145.5, 143.4, 137.0, 125.3, 121.8, 81.4, 56.6, 38.5, 37.4, 29.5, 24.3. **MS (m/z)**: calcd for $C_{12}H_{16}NO_3S$, $[M+H]^+$: 254.08; found, 254.2. **Melting point**: 86-88 °C.

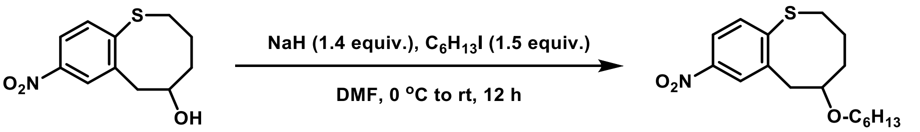

BT3

To a solution of the alcohol substrate (0.84 g, 3.5 mmol) in anhydrous DMF (35 mL) were added NaH (0.20 g, 4.9 mmol, 1.4 equiv.) and $C_6H_{13}I$ (1.11 g, 5.3 mmol, 1.5 equiv.) under 0 °C. After stirring at rt for overnight, the reaction mixture was quenched by H_2O (30 mL), then extracted with EtOAc (3×60 mL). The combined organic layers were washed with brine, dried over anhydrous Na_2SO_4 and concentrated *in vacuo*. The crude mixture was purified by column chromatography on silica gel (eluent: hexanes/ethyl acetate = 20:1) to give product **BT3** (white solid, 41% yield, 0.46 g).

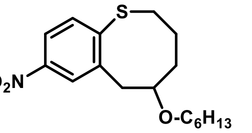

BT3

5-(Hexyloxy)-8-nitro-3,4,5,6-tetrahydro-2*H*-benzo[*b*]thiocine

^{1}H NMR (700 MHz, chloroform-*d*) δ 8.08 (d, *J* = 2.6 Hz, 1H), 7.99 (dd, *J* = 8.4, 2.6 Hz, 1H), 7.77 (d, *J* = 8.4 Hz, 1H), 3.63 (tt, *J* = 7.6, 3.8 Hz, 1H), 3.59 (dt, *J* = 9.0, 6.6 Hz, 1H), 3.51 – 3.43 (m, 2H), 3.39 (dd, *J* = 13.1, 7.2 Hz, 1H), 2.89 – 2.74 (m, 2H), 2.07 – 1.98 (m, 1H), 1.65 – 1.59 (comp, 3H), 1.57 – 1.53 (m, 1H), 1.49 – 1.43 (m, 1H), 1.42 – 1.36 (m, 2H), 1.35 – 1.29 (comp, 4H), 0.90 (t, *J* = 7.0 Hz, 3H). **^{13}C NMR** (176 MHz, chloroform-*d*) δ 148.0, 145.6, 143.4, 136.9, 125.4, 121.7, 79.7, 69.0, 38.6, 37.8, 31.9, 30.2, 30.1, 26.2, 24.5, 22.8, 14.2. **MS (m/z)**: calcd for $C_{17}H_{25}NNaO_3S$, $[M+Na]^+$: 346.14; found, 346.1. **Melting point**: 47-49 °C.

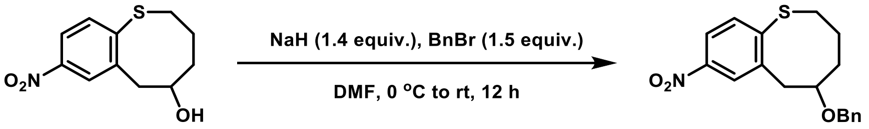

BT4

To a solution of the alcohol substrate (0.48 g, 2.0 mmol) in anhydrous DMF (20 mL) were added NaH (0.11 g, 2.8 mmol, 1.4 equiv.) and BnBr (0.51 g, 3.0 mmol, 1.5 equiv.) under 0 °C. After stirring at rt for overnight, the reaction mixture was quenched by H_2O (30 mL), then extracted with EtOAc (3×40 mL). The combined organic layers were washed with brine, dried over anhydrous Na_2SO_4 and concentrated *in vacuo*. The crude mixture was purified by column chromatography on silica gel (eluent: hexanes/ethyl acetate = 35:1) to give product **BT4** (white solid, 90% yield, 0.59 g).

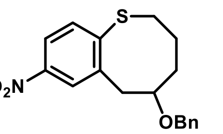

BT4

5-(Benzyloxy)-8-nitro-3,4,5,6-tetrahydro-2*H*-benzo[*b*]thiocine

^{1}H NMR (700 MHz, chloroform-*d*) δ 8.12 (d, *J* = 2.6 Hz, 1H), 8.00 (dd, *J* = 8.4, 2.6 Hz, 1H), 7.78 (d, *J* = 8.4 Hz, 1H), 7.42 – 7.36 (comp, 4H), 7.31 (tt, *J* = 6.2, 2.1 Hz, 1H), 4.68 (d, *J* = 11.9 Hz, 1H), 4.63 (d, *J* = 11.9 Hz, 1H), 3.79 (tt, *J* = 7.4, 4.0 Hz, 1H), 3.52 – 3.44 (m, 2H), 2.87 (ddd, *J* = 14.2, 8.5, 2.9 Hz, 1H), 2.81 (ddd, *J* = 14.2, 8.1, 2.9 Hz, 1H), 2.10 – 1.99 (m, 1H), 1.68 – 1.56 (comp, 3H). **^{13}C NMR** (176 MHz, chloroform-*d*) δ 148.0, 145.4, 143.4, 138.6, 136.9, 128.7, 127.8, 127.6, 125.4, 121.8, 79.1, 70.7, 38.5, 37.9, 30.0, 24.4. **MS (m/z)**: calcd for $C_{18}H_{20}NO_3S$, $[M+H]^+$: 330.12; found, 330.1. **Melting point**: 55-56 °C.

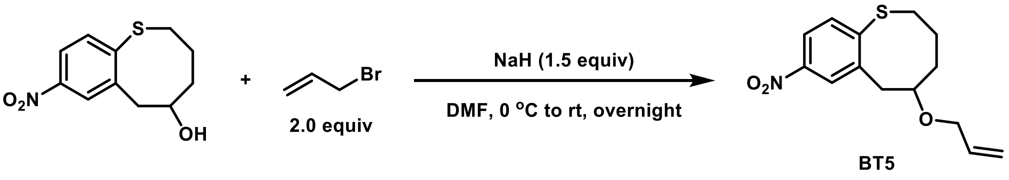

BT5

To a solution of the alcohol substrate (0.72 g, 3.0 mmol) in anhydrous DMF (20 mL) were added NaH (0.18 g, 4.5 mmol, 1.5 equiv.) and allyl bromide (0.73 g, 6.0 mmol, 2.0 equiv.) under 0 °C. After stirring at rt for overnight, the reaction mixture was quenched by H_2O (30 mL), then extracted with EtOAc (3×40 mL). The combined organic layers were washed with brine, dried over anhydrous Na_2SO_4 and concentrated *in vacuo*. The crude mixture was purified by column chromatography on silica gel (eluent: hexanes/ethyl acetate = 30:1) to give product **BT5** (white solid, 91% yield, 0.77 g).

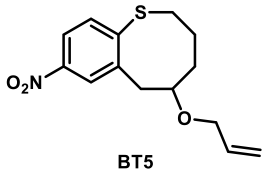

BT5

5-(Allyloxy)-8-nitro-3,4,5,6-tetrahydro-2*H*-benzo[*b*]thiocine

^{1}H NMR (700 MHz, chloroform-*d*) δ 8.09 (d, *J* = 2.6 Hz, 1H), 8.00 (dd, *J* = 8.4, 2.6 Hz, 1H), 7.78 (d, *J* = 8.4 Hz, 1H), 5.96 (ddt, *J* = 17.2, 10.4, 5.4 Hz, 1H), 5.34 (dt, *J* = 17.2, 1.6 Hz, 1H), 5.22 (dt, *J* = 10.4, 1.6 Hz, 1H), 4.14 (ddt, *J* = 12.8, 5.4, 1.6 Hz, 1H), 4.09 (ddt, *J* = 12.8, 5.4, 1.6 Hz, 1H), 3.72 (tt, *J* = 7.6, 3.9 Hz, 1H), 3.47 (dd, *J* = 13.1, 4.1 Hz, 1H), 3.42 (dd, *J* = 13.1, 7.3 Hz, 1H), 2.87 (ddd, *J* = 14.2, 8.5, 2.9 Hz, 1H), 2.81 (ddd, *J* = 14.2, 8.1, 2.9 Hz, 1H), 2.04 (dtd, *J* = 14.2, 7.9, 6.5, 3.3 Hz, 1H), 1.69 – 1.60 (m, 1H), 1.59 – 1.55 (m, 1H), 1.54 – 1.47 (m, 1H). **^{13}C NMR** (176 MHz, chloroform-*d*) δ 148.1, 145.5, 143.4, 136.9, 135.0, 125.4, 121.8, 117.0, 79.0, 69.6, 38.6, 37.8, 30.0, 24.4. **MS (m/z)**: calcd for $C_{14}H_{18}NO_3S$, $[M+H]^+$: 280.10; found, 280.2. **Melting point**: 52-53 °C.

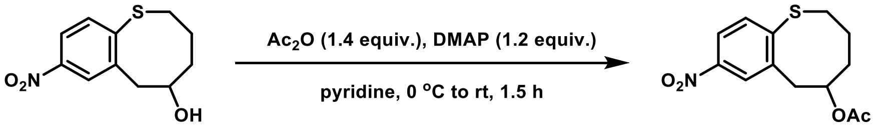

BT6

To a solution of the alcohol substrate (0.48 g, 2.4 mmol) in pyridine (20 mL) were added Ac_2O (0.34 g, 3.4 mmol, 1.4 equiv.) and DMAP (0.35 g, 2.9 mmol, 1.2 equiv.) under 0 °C. After stirring at rt for 1.5 h, the reaction mixture was quenched by H_2O (30 mL), then extracted with EtOAc (3×40 mL). The combined organic layers were washed with brine, dried over anhydrous Na_2SO_4 and concentrated *in vacuo*. The crude mixture was purified by column chromatography on silica gel (eluent: hexanes/ethyl acetate = 10:1) to give product **BT6** (white solid, 92% yield, 0.62 g).

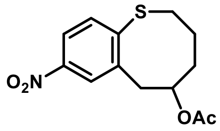

BT6

8-Nitro-3,4,5,6-tetrahydro-2*H*-benzo[*b*]thiocin-5-yl acetate

^{1}H NMR (700 MHz, chloroform-*d*) δ 8.11 (d, *J* = 2.5 Hz, 1H), 8.04 (dd, *J* = 8.4, 2.5 Hz, 1H), 7.82 (d, *J* = 8.4 Hz, 1H), 5.18 – 5.08 (m, 1H), 3.57 (dd, *J* = 13.4, 3.8 Hz, 1H), 3.41 (dd, *J* = 13.4, 7.2 Hz, 1H), 2.89 – 2.78 (m, 2H), 2.10 (s, 3H), 2.05 – 1.96 (m, 1H), 1.72 – 1.63 (m, 2H), 1.54 – 1.48 (m, 1H). **^{13}C NMR** (176 MHz, chloroform-*d*) δ 170.5, 148.2, 144.6, 143.3, 137.3, 125.4, 122.2, 74.1, 38.5, 37.7, 30.2, 24.5, 21.5. **MS (m/z)**: calcd for $C_{13}H_{16}NO_4S$, $[M+H]^+$: 282.08; found, 282.3. **Melting point**: 110-111 °C.

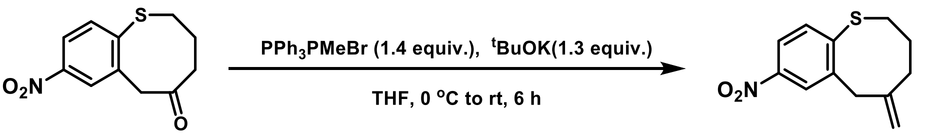

BT7

To a flask were added PPh3MeBr (1.0 g, 2.8 mmol, 1.4 equiv.), anhydrous THF (20 mL) and a stirring bar, and the system was cooled to 0 °C. Then ^{t}BuOK (0.29 g, 2.6 mmol, 1.3 equiv.) was

added in portions and the mixture was stirred for 30 min. The solution of **BT1** (0.48 g, 2.0 mmol, 1.0 equiv.) in anhydrous THF (10 mL) was added into the reaction mixture dropwise. After stirring at rt for 6 h, the reaction was quenched with H_2O (30 mL) and the mixture was extracted with DCM (3×30 mL). The combined organic layers were washed with brine, dried over anhydrous Na_2SO_4 and concentrated *in vacuo*. The crude mixture was purified by column chromatography on silica gel (eluent: hexanes/ethyl acetate = 12:1) to give product **BT7** (white solid, 60% yield, 0.28 g).

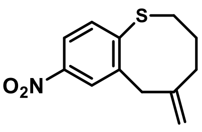

BT7

5-Methylene-8-nitro-3,4,5,6-tetrahydro-2*H*-benzo[*b*]thiocine

^{1}H NMR (700 MHz, chloroform-*d*) δ 8.10 (d, *J* = 2.6 Hz, 1H), 7.99 (dd, *J* = 8.4, 2.6 Hz, 1H), 7.77 (d, *J* = 8.4 Hz, 1H), 4.98 – 4.95 (m, 1H), 4.78 – 4.74 (m, 1H), 3.93 (s, 2H), 2.90 – 2.86 (m, 2H), 2.13 – 2.09 (m, 2H), 1.89 – 1.83 (m, 2H). **^{13}C NMR** (176 MHz, chloroform-*d*) δ 149.0, 148.4, 147.7, 142.6, 137.0, 124.3, 121.8, 112.7, 42.7, 38.9, 34.6, 29.4. **MS (m/z)**: calcd for $C_{12}H_{14}NO_2S$, $[M+H]^+$: 236.07; found, 236.1. **Melting point**: 86-87 °C.

3. Optimization of the polymerization conditions

Table S1. Investigation of solvents for the polymerization[a]

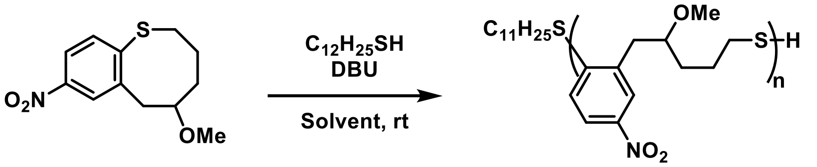

entry	solvent	concentration (M)	time (min)	conversion (%)[b]	$M_{n,SEC}$ (kDa)[c]	$Đ$[c]
1	DMSO	1.0	60	95	21.7	1.73
2	DMF	1.0	60	53	6.4	1.53
3	DMF	1.5	270	77	15.1	1.58
4	DMA	1.5	120	95	11.0	1.78
5	NMP	1.5	90	95	10.9	1.76
6	DMPU	1.5	150	95	12.6	1.80
7	CH_3CN[d]	1.0	200	59	9.9	1.70
8	$PhNO_2$	2.0	300	42	4.8	1.46
9	$CHCl_3$	1.0	60	0	-	-
10	DCE	2.0	360	0	-	-
11	THF	1.0	60	0	-	-

[a]$[M]_0/[I]_0/[base]_0$ = 50/1/1. [b]Conversions were determined by ^{1}H NMR spectroscopic analysis of the reaction mixture. [c]Molecular weights ($M_{n,SEC}$)and dispersities ($Đ$)were determined by size-exclusion chromatography. [d]40 °C

Table S2. Investigation of temperature and concentration for the polymerization[a]

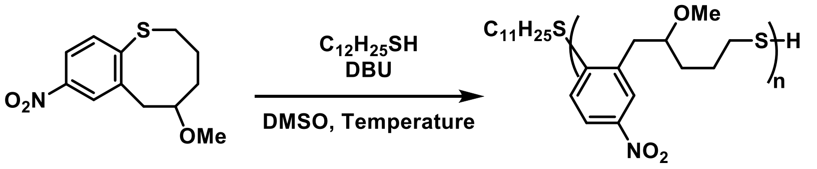

entry	temperature (℃)	concentration (M)	time (min)	conversion (%)[b]	$M_{n,SEC}$ (kDa)[c]	$Đ^c$
1	40	1.25	60	95	16.4	1.66
2	60	1.60	60	92	18.3	1.66
3	90	3.5	60	94	24.3	1.57
4	90	4.0	60	92	24.0	1.76
5	90	5.0	180	48	16.9	1.58

[a]$[M]_0/[I]_0/[base]_0 = 50/1/1$. [b]Conversions were determined by ^{1}H NMR spectroscopic analysis of the reaction mixture. [c]Molecular weights ($M_{n,SEC}$)and dispersities ($Đ$)were determined by size-exclusion chromatography.

Table S3. Further investigation of solvents for the polymerization at melting condition[a]

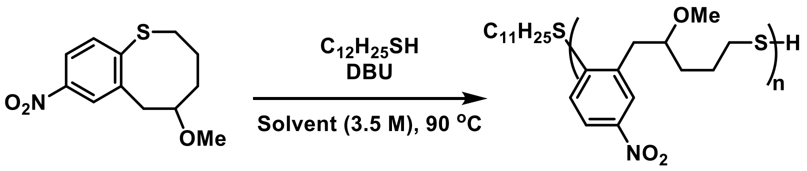

entry	solvent	time (min)	conversion (%)[b]	$M_{n,SEC}$ (kDa)[c]	$Đ^c$
1	DMF	120	92	16.4	1.51
2	NMP	120	91	12.5	1.74
3	NMC	180	83	12.4	1.70
4	$PhNO_2$	180	42	12.0	1.23
5	PhCN	180	46	7.8	1.51
6	PC	300	16	2.8	1.74

[a]$[M]_0/[I]_0/[base]_0 = 50/1/1$. [b]Conversions were determined by ^{1}H NMR spectroscopic analysis of the reaction mixture. [c]Molecular weights ($M_{n,SEC}$)and dispersities ($Đ$)were determined by size-exclusion chromatography. DMF: dimethylformamide. NMP: *N*-methyl-2-pyrrolidone. NMC: *N*-methylcaprolactam. PC: propylene carbonate.

Table S4. Investigation of initiators for the polymerization[a]

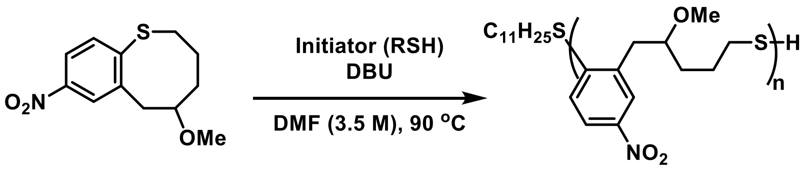

entry	Initiator (RSH)	pK_a^{DMSO}	time (min)	conversion (%)[b]	$M_{n,SEC}$ (kDa)[c]	$Đ^c$
1	BnSH	15.4	210	82	16.6	1.61
2	BnSH[d]	15.4	120	0	-	-
3	PhSH	10.3	180	19	4.3	1.39
4	$C_{12}H_{25}SH$	17.9	120	92	16.4	1.51

[a]$[M]_0/[I]_0/[base]_0 = 50/1/1$. [b]Conversions were determined by ^{1}H NMR spectroscopic analysis of the reaction mixture. [c]Molecular weights ($M_{n,SEC}$)and dispersities ($Đ$)were determined by size-exclusion chromatography. [d]Et_3N as the base.

Table S5. Investigation of bases for the polymerization[a]

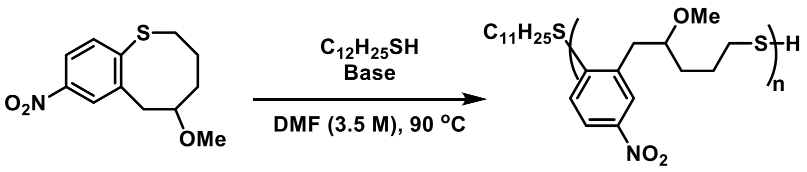

entry	base	$pK_a^{CH_3CN}$	time (min)	conversion (%)[b]	$M_{n,SEC}$ (kDa)[c]	$Đ$[c]
1	BTPP[d]	28	15	96	17.7	1.61
2	Et_3N	18.46	120	0	-	-
3	DMAP	17.95	120	0	-	-
4	DBU	24.34	120	92	16.4	1.51

[a]$[M]_0/[I]_0/[base]_0$ = 50/1/1. [b]Conversions were determined by ^{1}H NMR spectroscopic analysis of the reaction mixture. [c]Molecular weights ($M_{n,SEC}$)and dispersities ($Đ$)were determined by size-exclusion chromatography. [d]Phosphazene base P1-*t*-Bu-tris(tetramethylene)

4. General polymerization procedure and characterization of polymers

Preparation of stock solution: The desired amounts of 1-dodencanethiol and BTPP were added into an oven-dried 2 mL vial under N_2. Dry, degassed DMF was then added to make a stock solution.

To an oven-dried microwave vial equipped with a magnetic stir bar was added the benzothiocane monomer (0.3 mmol). After evacuation and backfilling with N_2 three times, the vial was placed into a preheated oil bath. Dry, degassed DMF and the initiator stock solution were added to the vial. After stirring for the indicated time, the reaction was quenched by three drops of trifluoracetic acid or the solution of iodoacetamide (5.55 mg, 0.03 mmol, 10 equivalents to initiator) in $CDCl_3$ and cooled to room temperature. An aliquot of the reaction mixture was taken for ^{1}H NMR to determine the conversion of the monomer. Another aliquot of the reaction mixture was taken for SEC analysis. The resulting polymer was precipitated from cold MeOH and further purified by precipitating into MeOH/DCM (1:2.5). The purified polymer was then characterized using SEC, ^{1}H-NMR, ^{13}C-NMR, TGA and DSC.

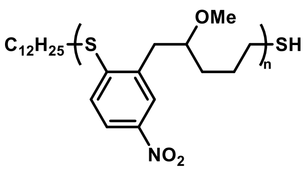

PBT2

^{1}H NMR (700 MHz, chloroform-*d*) δ 8.06 – 7.96 (comp, 2H), 7.27 (d, *J* = 8.8 Hz, 1H), 3.51 (p, *J* = 6.2 Hz, 1H), 3.30 (s, 3H), 3.08 – 2.97 (comp, 3H), 2.79 (dd, *J* = 14.1, 6.0 Hz, 1H), 1.96 – 1.86 (m, 1H), 1.84 – 1.73 (m, 1H), 1.72 – 1.60 (m, 2H). **^{13}C NMR** (176 MHz, chloroform-*d*) δ 146.9, 144.8, 137.3, 125.2, 125.1, 122.1, 79.3, 57.5, 38.1, 33.2, 32.5, 24.5.

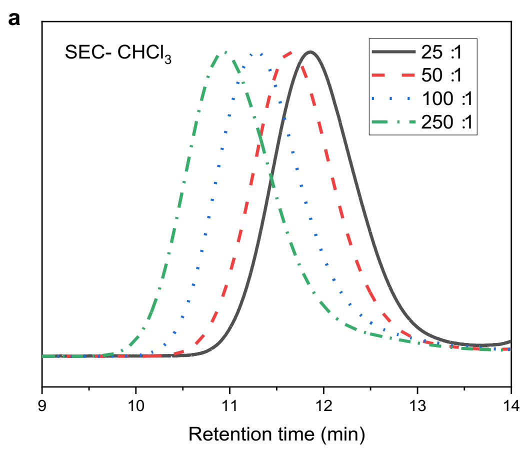

Figure S1. SEC trace for **PBT2s** targeting different DP.

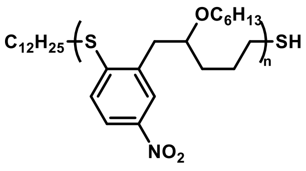

^{1}H NMR (700 MHz, chloroform-*d*) δ 8.06 – 7.99 (comp, 2H), 7.28 – 7.26 (m, 1H), 3.57 (p, *J* = 6.3 Hz, 1H), 3.41 – 3.34 (m, 1H), 3.33 – 3.27 (m, 1H), 3.09 – 2.95 (comp, 3H), 2.82 (dd, *J* = 14.1, 5.7 Hz, 1H), 1.98 – 1.87 (m, 1H), 1.84 – 1.74 (m, 1H), 1.72 – 1.61 (m, 2H), 1.44 (p, *J* = 6.9 Hz, 2H), 1.28 – 1.11 (comp, 6H), 0.83 (t, *J* = 7.1 Hz, 3H). **^{13}C NMR** (176 MHz, chloroform-*d*) δ 146.8, 144.7, 137.6, 125.4, 125.0, 122.0, 77.7, 70.1, 38.7, 33.8, 32.5, 31.8, 30.2, 26.0, 24.6, 22.7, 14.2.

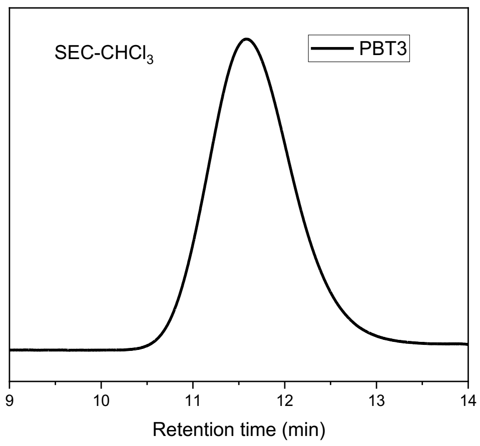

Figure S2. SEC trace of polymer **PBT3**.

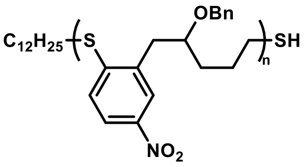

^{1}H NMR (700 MHz, chloroform-*d*) δ 8.08 – 7.88 (comp, 2H), 7.29 – 7.10 (m, 6H), 4.48 – 4.37 (m, 2H), 3.73 (p, *J* = 6.0 Hz, 1H), 3.11 – 3.02 (m, 1H), 2.95 (t, *J* = 7.3 Hz, 2H), 2.85 (dd, *J* = 14.1, 5.8 Hz, 1H), 1.95 – 1.84 (m, 1H), 1.81 – 1.74 (m, 1H), 1.73 – 1.64 (m, 2H). **^{13}C NMR** (176 MHz, chloroform-*d*) δ 146.7, 144.7, 138.1, 137.3, 128.5, 128.0, 127.9, 125.5, 125.1, 122.1, 77.0, 71.8, 38.7, 33.5, 32.4, 24.3.

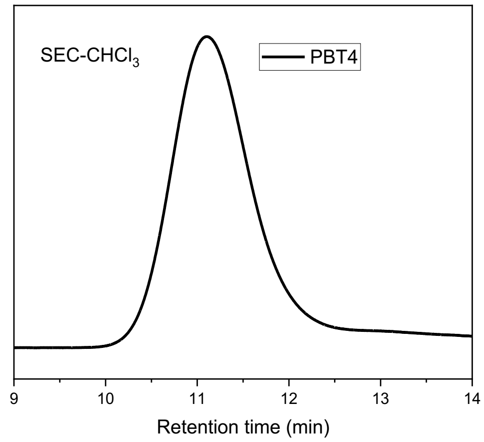

Figure S3. SEC trace of polymer **PBT4**.

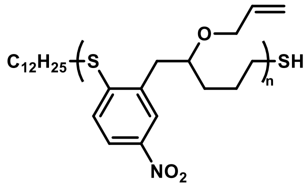

PBT5

[1]**H NMR** (700 MHz, chloroform-*d*) δ 8.06 – 7.98 (comp, 2H), 7.27 (d, *J* = 8.1 Hz, 1H), 5.76 (ddt, *J* = 16.7, 10.6, 5.6 Hz, 1H), 5.17 (dd, *J* = 16.7, 1.8 Hz, 1H), 5.09 (dd, *J* = 10.6, 1.8 Hz, 1H), 3.92 (d, *J* = 5.6 Hz, 2H), 3.66 (p, *J* = 6.2 Hz, 1H), 3.09 – 2.97 (comp, 3H), 2.83 (dd, *J* = 14.1, 6.0 Hz, 1H), 1.97 – 1.88 (m, 1H), 1.85 – 1.73 (m, 1H), 1.73 – 1.63 (m, 2H). [13]**C NMR** (176 MHz, chloroform-*d*) δ 146.8, 144.7, 137.3, 134.7, 125.4, 125.1, 122.1, 117.2, 70.8, 38.7, 33.6, 32.5, 24.5.

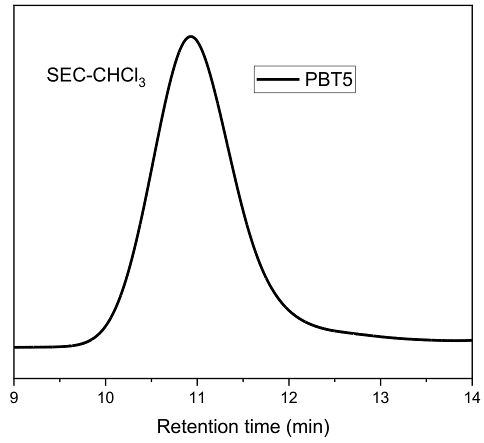

Figure S4. SEC trace of polymer **PBT5**.

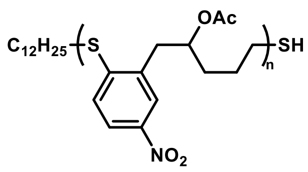

PBT6

[1]**H NMR** (700 MHz, chloroform-*d*) δ 8.11 – 7.84 (comp, 2H), 7.25 (d, *J* = 8.7 Hz, 1H), 5.25 – 5.08 (m, 1H), 3.12 – 2.95 (comp, 3H), 2.96 – 2.86 (m, 1H), 1.93 (s, 3H), 1.84 – 1.68 (comp, 4H). [13]**C NMR** (176 MHz, chloroform-*d*) δ 170.5, 146.8, 144.7, 136.2, 125.4, 125.2, 122.4, 71.6, 38.4, 33.6, 32.1, 24.4, 21.1.

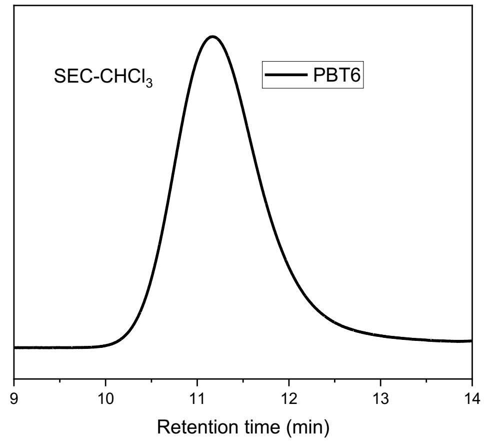

Figure S5. SEC trace of polymer **PBT6**.

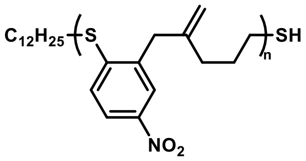

PBT7

^{1}H NMR (700 MHz, chloroform-*d*) δ 8.07 – 7.99 (m, 1H), 7.97 – 7.89 (m, 1H), 7.27 (d, *J* = 8.8 Hz, 1H), 4.93 (s, 1H), 4.67 (s, 1H), 3.43 (s, 2H), 3.01 (t, *J* = 7.4 Hz, 2H), 2.21 (t, *J* = 7.4 Hz, 2H), 1.89 (p, *J* = 7.4 Hz, 2H). **^{13}C NMR** (176 MHz, chloroform-*d*) δ 147.2, 144.9, 144.5, 138.0, 125.2, 124.5, 122.1, 113.8, 39.9, 35.0, 31.7, 26.4.

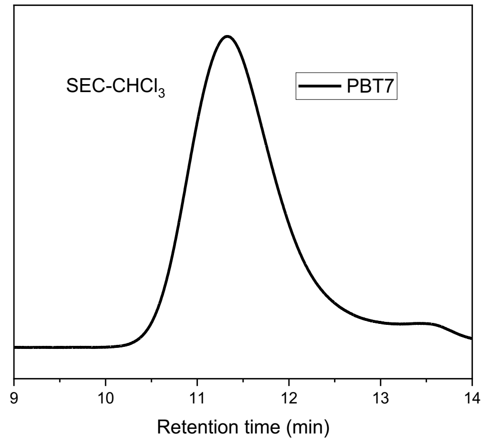

Figure S6. SEC trace of polymer **PBT7**.

5. Model S$_N$Ar exchange reaction

Under nitrogen atmosphere, to an oven-dried NMR tube were added aryl dodecyl sulfide (1.0 equiv.), benzyl mercaptan (1.0 equiv.), K_2CO_3 (1.5 equiv.) and dry d_6-DMSO. The reaction was monitored by ^{1}H NMR spectroscopy.

a) Exchange reaction between 4-(dodecylthio)benzonitrile and benzyl mercaptan:

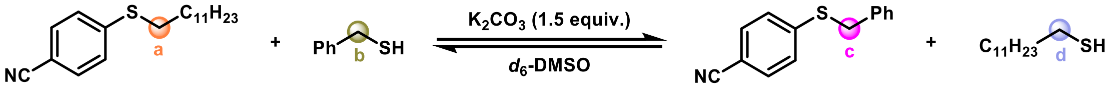

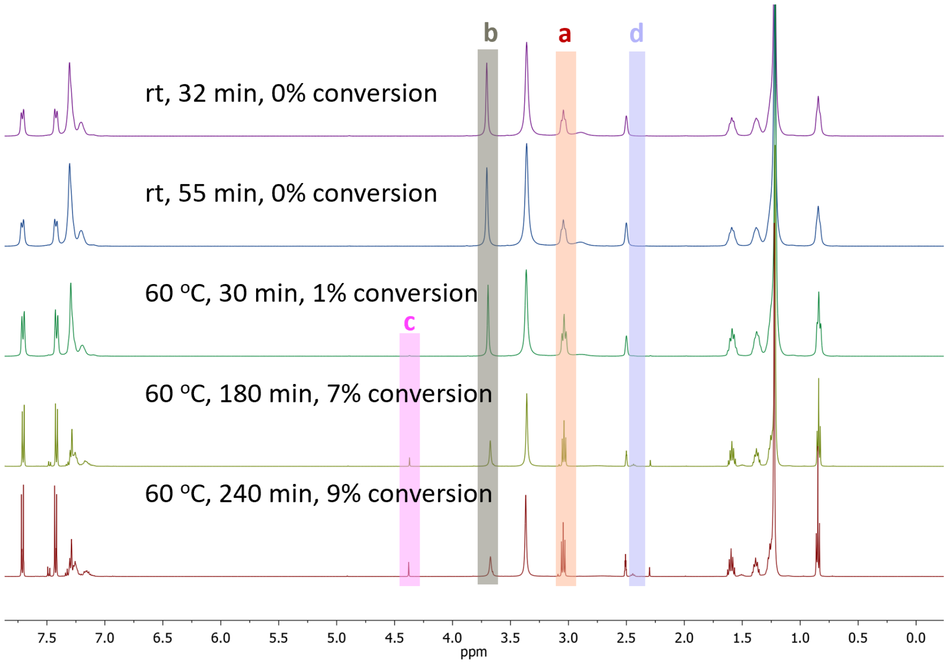

Figure S7. Overlay of ^{1}H-NMR spectra of exchange reaction between 4-(dodecylthio)benzonitrile and benzyl mercaptan.

b) Exchange reaction between 4-(dodecylthio)benzaldehyde and benzyl mercaptan:

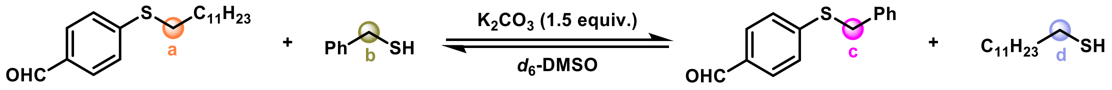

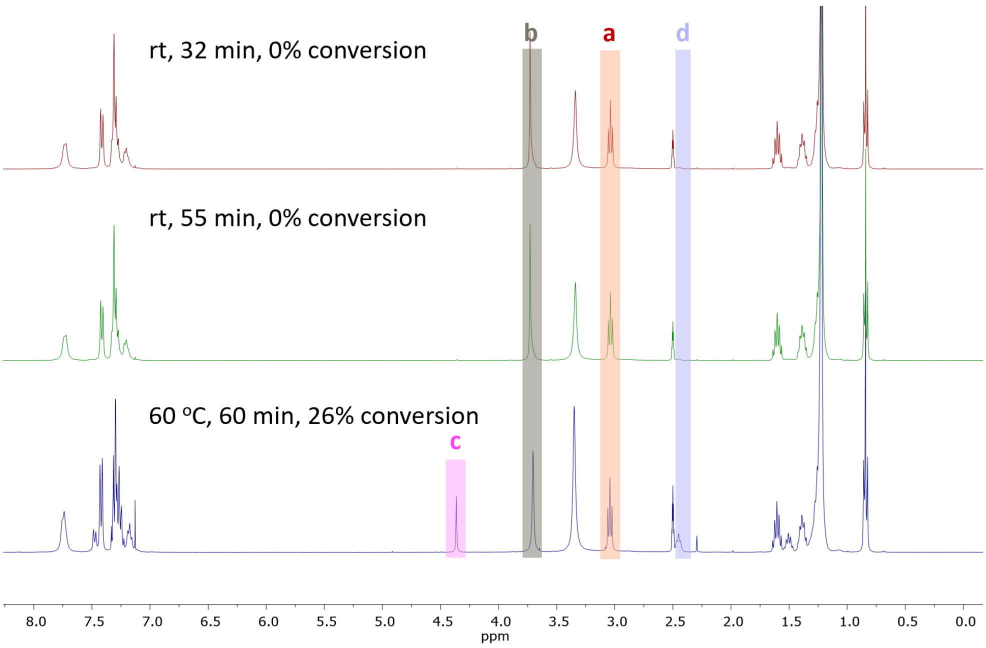

Figure S8. Overlay of ^{1}H-NMR spectra of exchange reaction between 4-(dodecylthio)benzaldehyde and benzyl mercaptan.

c) Exchange reaction between dodecyl(4-nitrophenyl)sulfane and benzyl mercaptan:

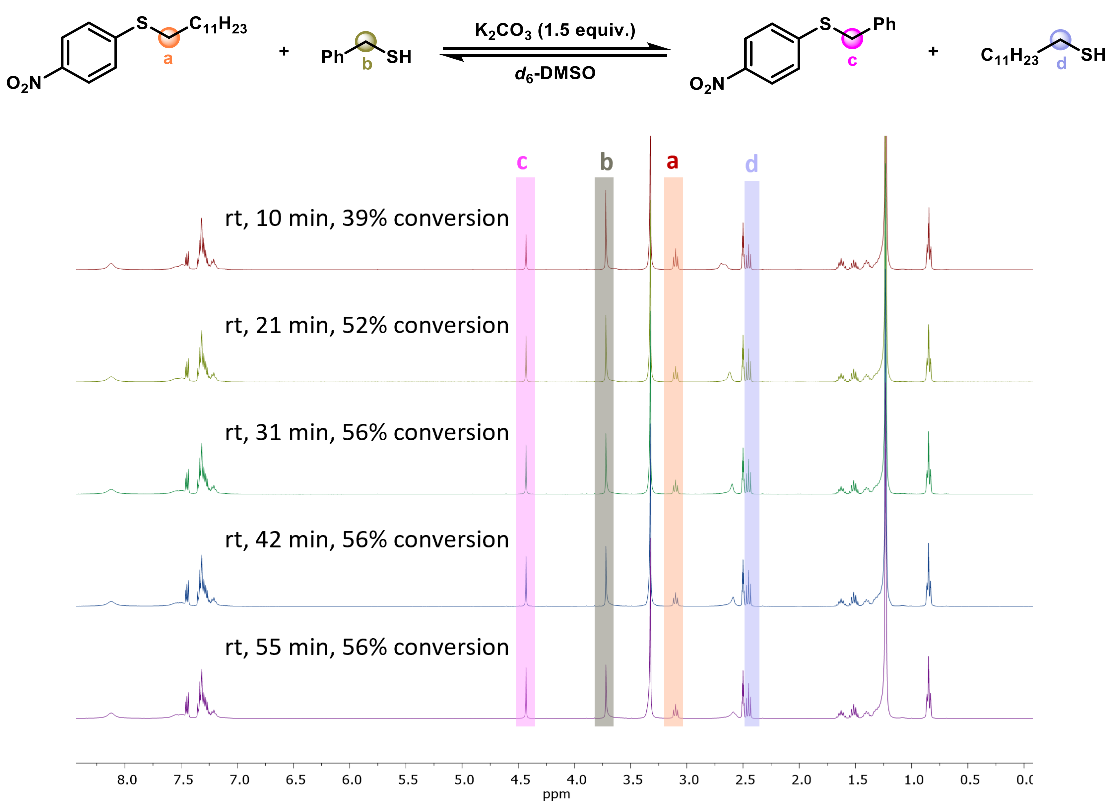

Figure S9. Overlay of ^{1}H-NMR spectra of exchange reaction between dodecyl(4-nitrophenyl)sulfane and benzyl mercaptan.

d) Exchange reaction between BT1 and benzyl mercaptan:

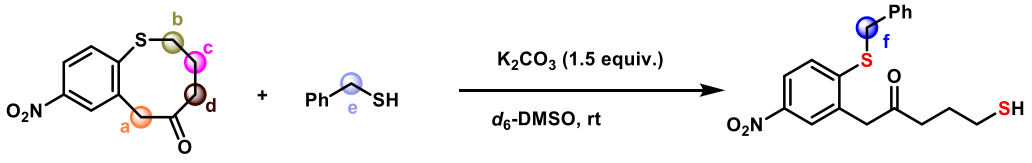

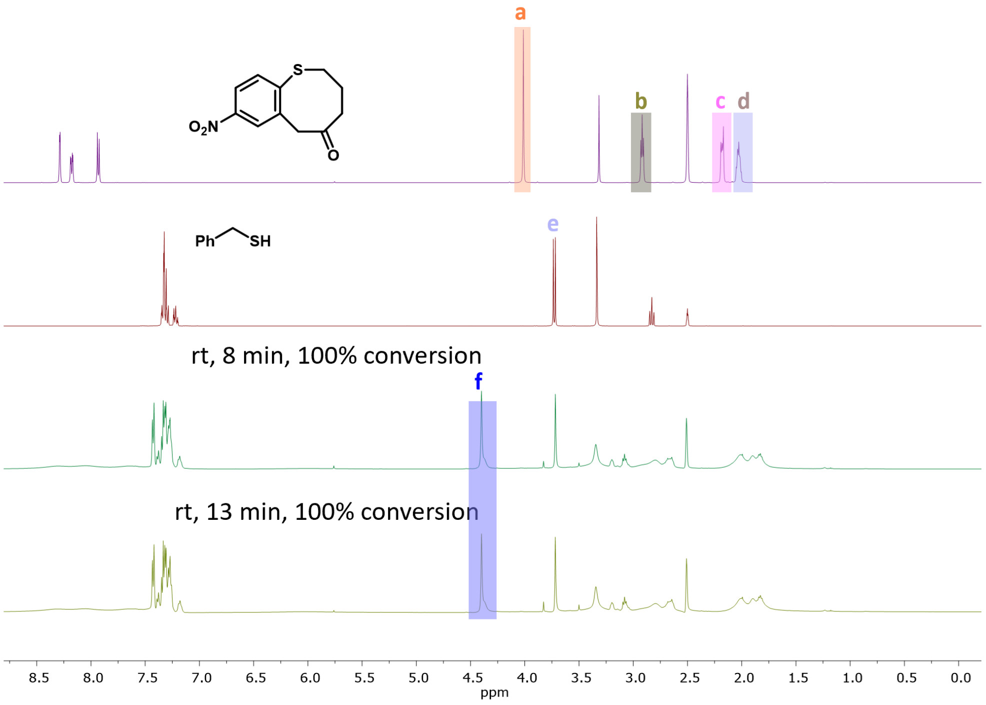

Figure S10. Overlay of ^{1}H-NMR spectra of exchange reaction between **BT1** and benzyl mercaptan.

e) Exchange reaction between BT8 and benzyl mercaptan:

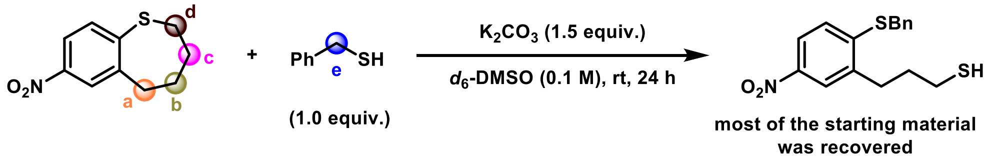

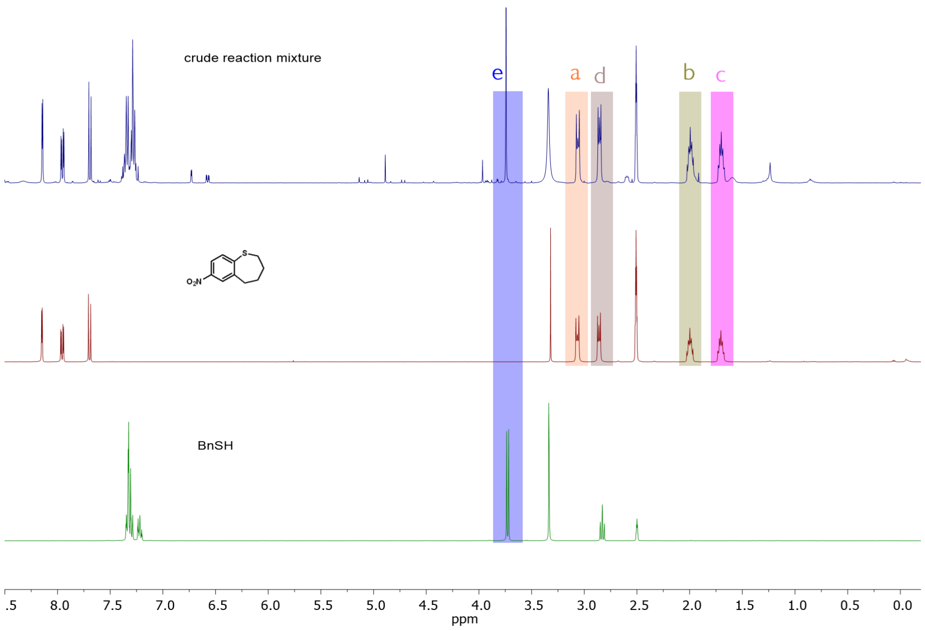

Figure S11. Overlay of ^{1}H-NMR spectra of exchange reaction between **BT8** and benzyl mercaptan.

6. Analysis of cyclic oligomer by-products and the linear structure of PBT

Example polymerization of BT2 targeting DP100:

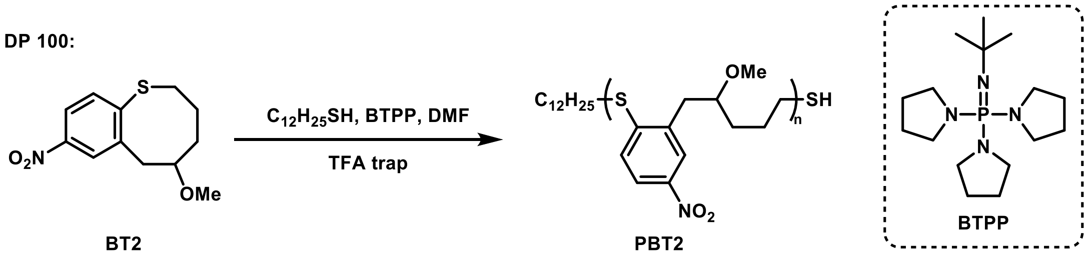

Preparation of stock solution: 1-dodencanethiol (18.2 mg, 21.5 μL, 0.09 mmol) and BTPP (28.1mg, 27.5 μL, 0.09 mmol) were added into an oven-dried 2 mL vial under N_2. Then dry DMF (1031 μL) was added to make a stock solution.

To an oven-dried microwave vial equipped with a magnetic stir bar was added the benzothiocane monomer (0.3 mmol). After evacuation and backfilling with N_2 three times, the vial was placed into a preheated oil bath. The initiator stock solution (36 μL) was added to the monomer solution. The total volume is around 86 μL and the initiating concentration of the monomer $[M]_0$ is around 3.5 M. After stirring for 25 min, the reaction was quenched by three drops of trifluoracetic acid and cooled to room temperature. A sample of the crude material was taken for ^{1}H NMR to determine the conversion of the monomer. Another small amount of the crude material was taken for SEC analysis. The product was firstly purified by precipitating from cold methanol (10 mL). The mother liquor was concentrated and analyzed by matrix-assisted laser desorption/ionization-time-of-flight (MALDI-TOF) mass spectrometry. The precipitation was further purified by slowing adding MeOH (0.8 mL) into the crude product DCM solution (2.0 mL) with vigorous stirring. The overlay of the SEC traces from crude reaction mixture, 1st purification and 2nd purification were shown in Figure S11.

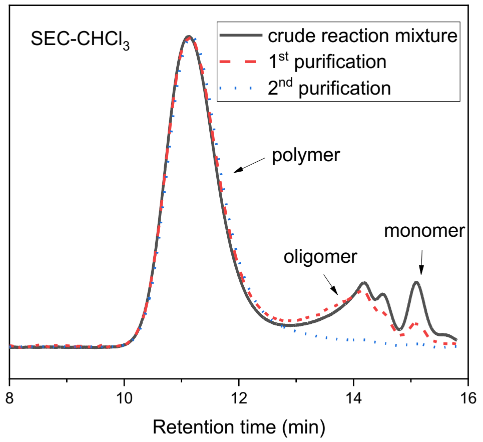

Figure S12. SEC curves for crude reaction mixture, product from the 1st purification and product from the 2nd purification from S_NArROP of **BT2** targeting DP 100.

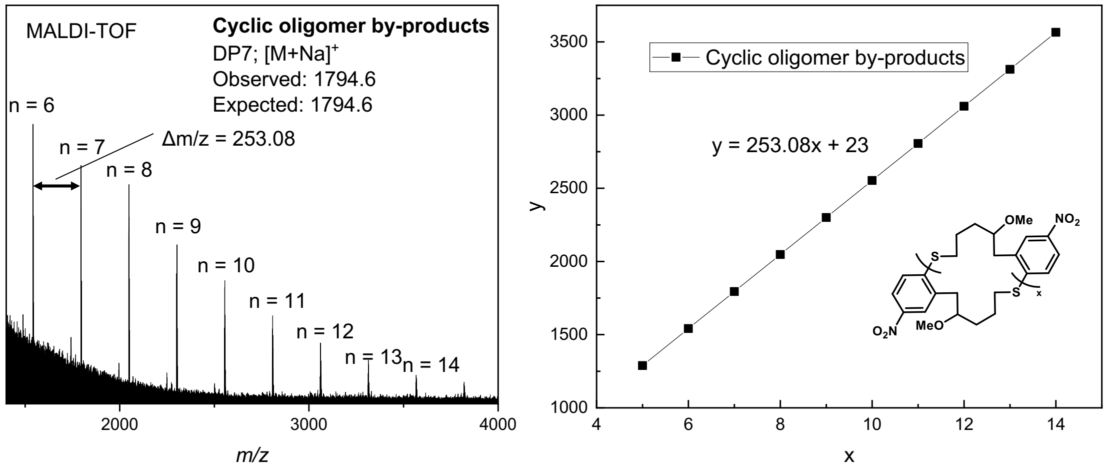

Figure S13. MALDI-TOF of cyclic oligomer by-products.

The linear structure of PBT: MALDI-TOF of **PBT2** (DP25)

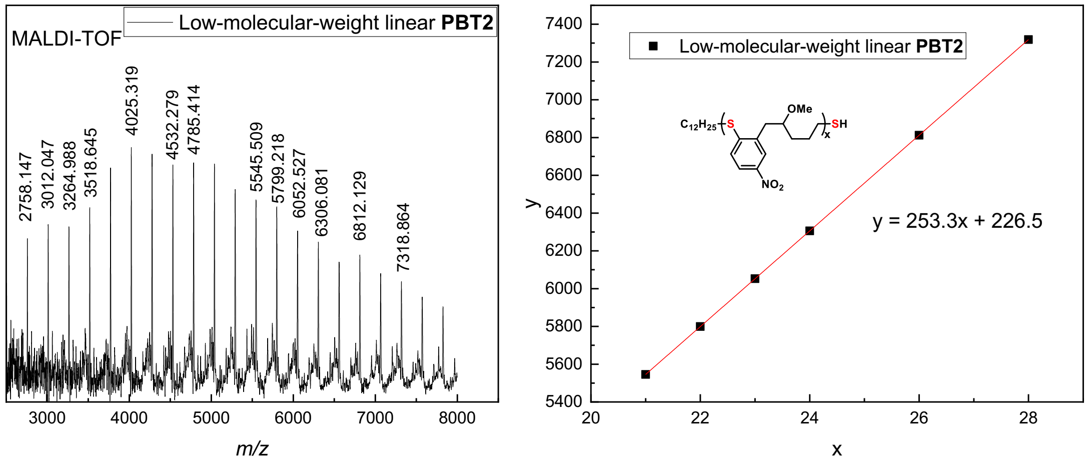

Figure S14. MALDI-TOF MS spectrum (left) of the low-molecular-weight linear **PBT2** (DP25) and linear plot (right) of *m/z* values (y) vs the number of **BT2** repeat units (x).

The linear structure of PBT: *^{1}H-NMR analysis of the product PBT2 (DP50) revealed that the triplet methyl group belongs to the chain-end -$C_{12}H_{25}$ (DP ≈43 calculated from integration).*

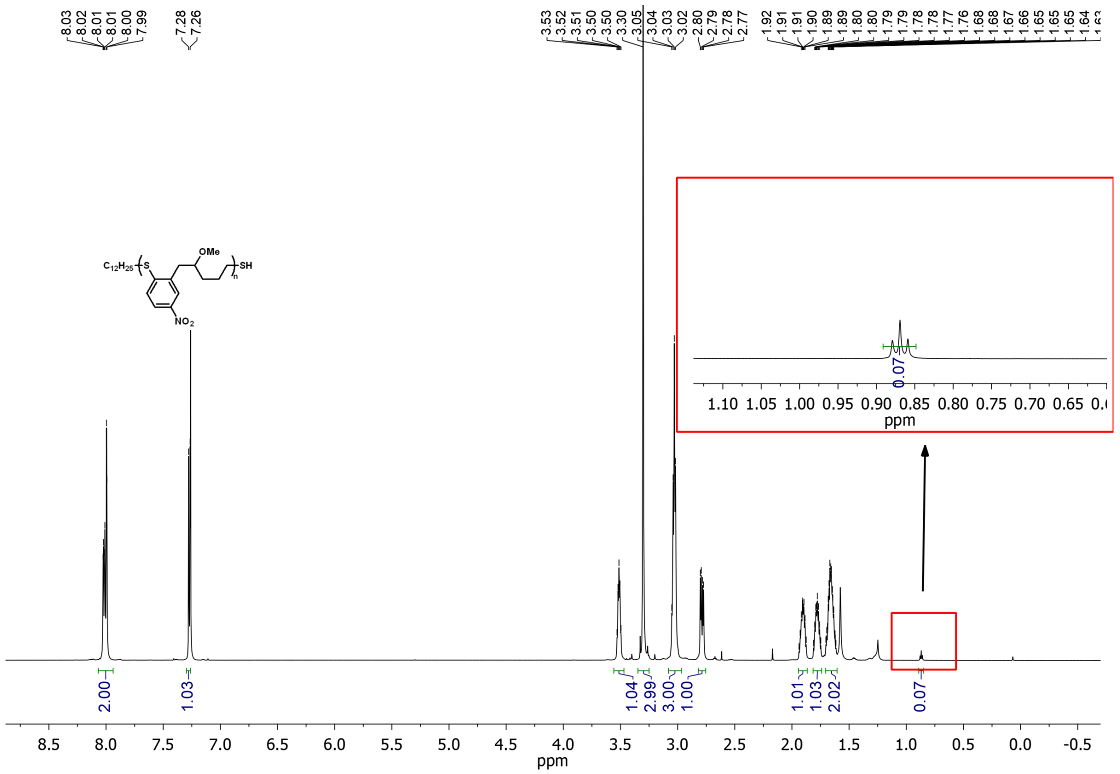

Figure S15. ^{1}H-NMR spectrum (left) of **PBT2** (DP50).

7. TGA and DSC Studies of the PBTs

TGA of purified **PBTs** were obtained in a nitrogen atmosphere at a heating rate of 10 °C/min. The $T_{d,5\%}$ (temperature causing a 5% weight loss) of each **PBT** was listed in Figure S9a.

DSC analysis of purified **PBTs** were performed (-20 °C to 250 °C, heating rate: 10 °C/min, cooling rate: 10 °C/min). The glass transition temperature (T_g) of each **PBT** was listed in Figure S9b.

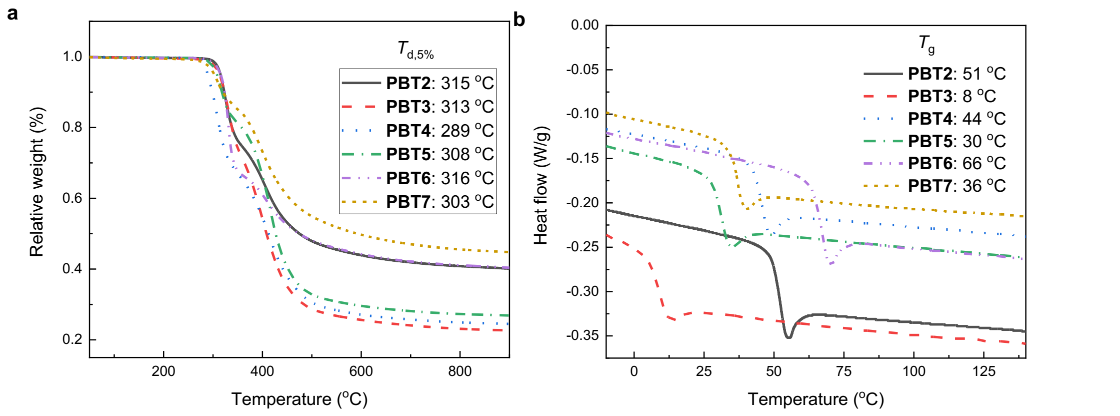

Figure S16. Physical properties of **PBTs** a, TGA curves for **PBT2-PBT7**. b, DSC curves for for **PBT2-PBT7**.

8. Mechanical properties of the PBT2s

PBT2s with two different M_n (39.7 kDa and 101.9 kDa) were prepared according to the general polymerization procedure. The **PBT2s**(1g) were dissolved in DCM (2g) and poured into a PTFE mold, then dried under ambient conditions for 8 hours. The sample was subsequently hot

compressed at 120°C for 20 minutes, maintaining a controlled thickness of 1mm.. Then, the samples were cut into strips (5mm x 30mm) for testing. Their thermomechanical properties were measured on a dynamic mechanical analysis tester (Q800, TA Instruments, New Castle, DE) with a frequency of 1 Hz in a tension mode with a temperature ramp 10 °C/min. The uniaxial tension tests were performed with a universal test machine (Insight 10, MTS Systems Corp., Eden Prairie, MN, USA) with a cross-head speed of 5 mm/min.

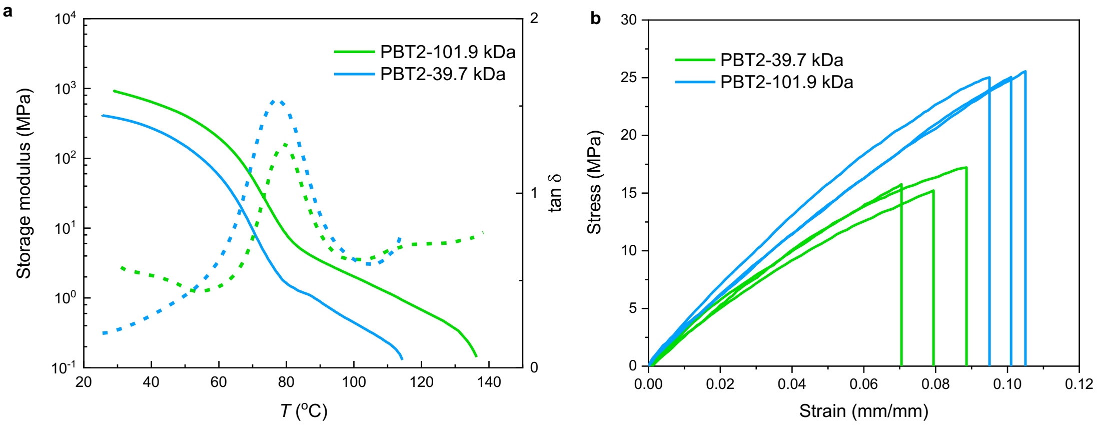

Figure S17. Mechanical properties of PBT2s. **a**, DMA storage modulus and tanδ profiles of **PBT2s** with different molecular weights; **b**, Tensile stress-strain curves of **PBT2s** with different molecular weights.

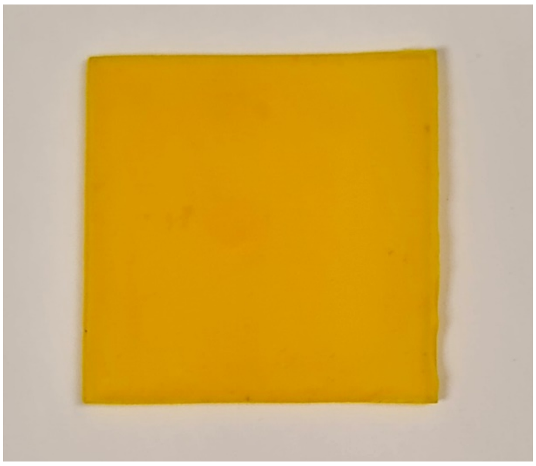

Figure S18. The photograph of PBT2 (20*20*1 mm).

9. Chemical recycle of PBTs

a) Depolymerization of PBT2:

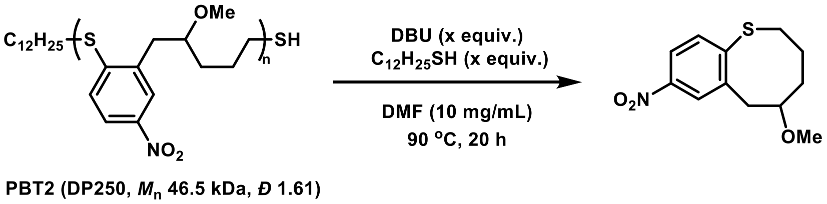

To a solution of **PBT2** (DP250, 4.65 mg, 10^{-4} mmol, 1.0 equiv.; repeat unit: 1.83×10^{-2} mmol) in DMF was added the stock solution of DBU and $C_{12}H_{25}SH$ in DMF. After stirring for 20 h under

90 °C, the reaction was quenched by TFA. The solvent was removed by evaporation and the residue was analyzed by ^{1}H NMR spectroscopy and SEC. The crude mixture from the conditions of 0.55 equiv. DBU and $C_{12}H_{25}SH$ was purified by column chromatography on silica gel (eluent: hexanes/ethyl acetate = 12:1) to give product **BT2** (85% yield, 3.96 mg).

Table S6. Investigation of the amount of DBU and $C_{12}H_{25}SH$ for the depolymerization[a]

entry	DBU	$C_{12}H_{25}SH$	conversion (%)[b]
1	0.0274 equiv.	0.0274 equiv.	trace
2	0.0548 equiv.	0.0548 equiv.	18
3	0.137 equiv.	0.137 equiv.	49
4	0.274 equiv.	0.274 equiv.	73
5	0.55 equiv.	0.55 equiv.	85 (85)[c]

[a]The amount of DBU and $C_{12}H_{25}SH$ was relative to the repeat unit. [b]The conversion was determined by ^{1}H NMR spectroscopic analysis of the reaction mixture. [c]Isolated yield of recovered monomer in parentheses.

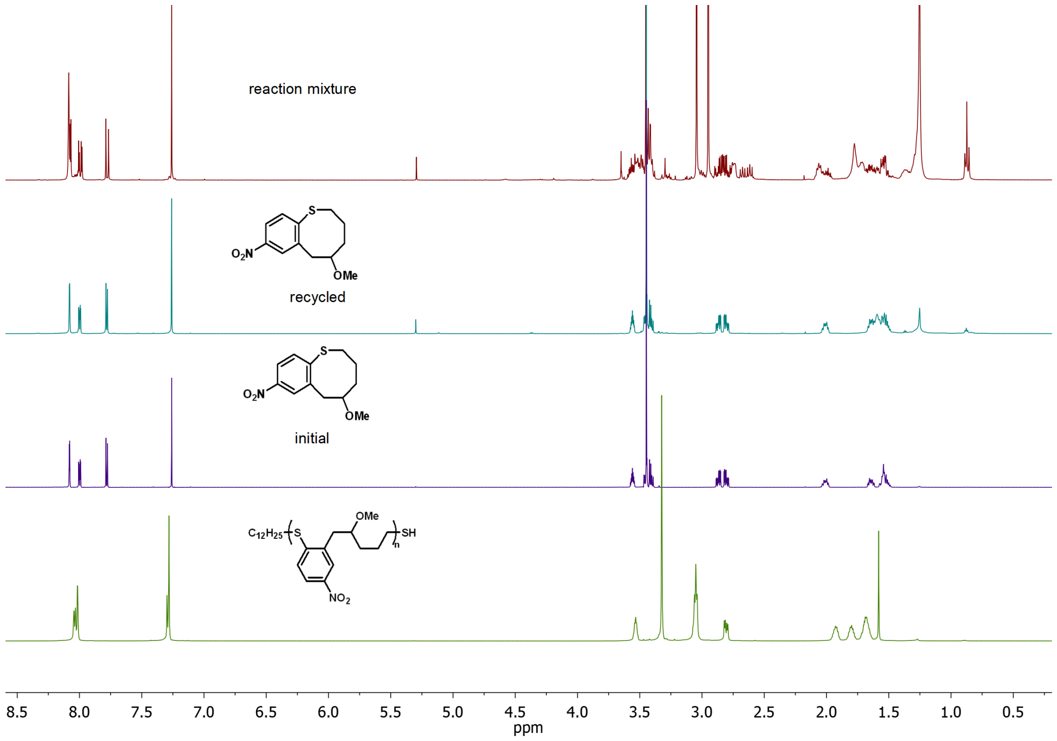

Figure S19. Overlay of ^{1}H-NMR spectra of the reaction mixture under the optimized depolymerization conditions, recycled and initial **BT2** and **PBT2**.

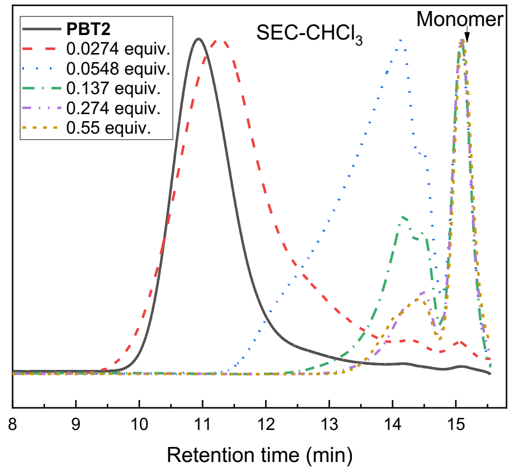

Figure S20. SEC curves for depolymerization of **PBT2** (46.5 kDa) with different amounts of DBU and $C_{12}H_{25}SH$ from 0.0274 equiv to 0.55 equiv (relative to the repeat unit).

b) Depolymerization of PBT5 (capped with iodoacetamide):

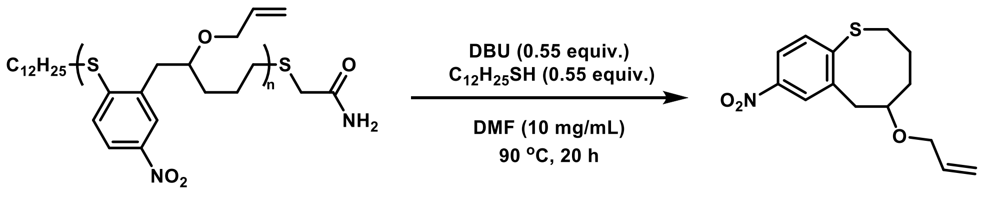

To a solution of **PBT5** (DP100, 9.9 mg, 2×10^{-4} mmol, 1.0 equiv.; repeat unit: 3.54×10^{-2} mmol) in DMF was added the stock solution of DBU and $C_{12}H_{25}SH$ in DMF. After stirring for 20 h under 90 ℃, the reaction was quenched by TFA. The solvent was removed by evaporation and the residue was analyzed by ^1H NMR spectroscopy and then purified by column chromatography on silica gel (eluent: hexanes/ethyl acetate = 12:1) to give product **BT5** (70% yield, 7.0 mg).

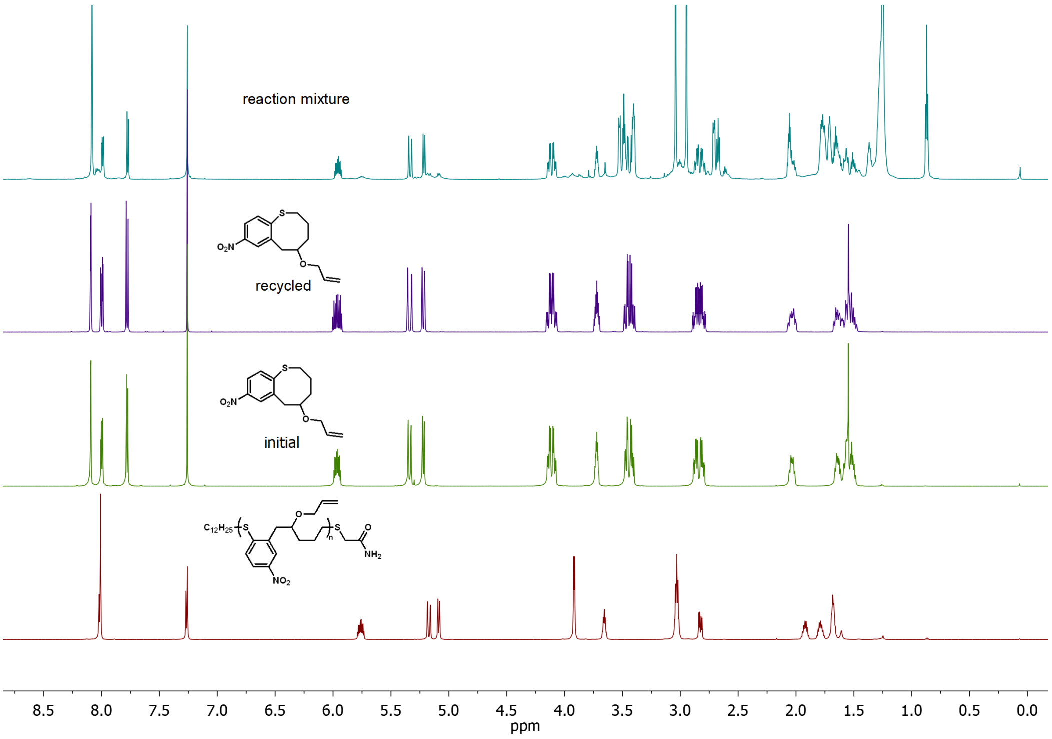

Figure S21. Overlay of ^{1}H-NMR spectra of the reaction mixture under the optimized depolymerization conditions, recycled and initial **BT2** and **PBT2**.

10. Polymerization thermodynamic studies of BT2

A representative procedure for the polymerization thermodynamic studies is shown as follows.
Preparation of stock solution: 1-dodencanethiol (20.24 mg, 24.0 μL, 0.1 mmol) and DBU (15.22 mg, 15.0 μL, 0.1 mmol) were added into an oven-dried 2 mL vial under N_2. Then dry DMSO (211 μL) was added to make a stock solution.
To four oven-dried microwave vials equipped with magnetic stir bars were added the benzothiocane monomer (0.2 mmol in each vial). After evacuation and backfilling with N_2 three times, 750 μL DMSO was added into each vial. The vials were then placed into a preheated oil bath at different temperatures, including 31°C, 60 °C, 90 °C and 120 °C. The initiator stock solution (20 μL) was added to the monomer solution. According to the kinetic studies, all polymerizations at room temperature reached the equilibrium by 60 min, polymerizations at higher temperatures should require shorter time. After stirring for 60 min, the reaction was quenched by three drops of trifluoracetic acid and cooled to room temperature. The monomer concentration at the equilibrium was determined by ^{1}H NMR.

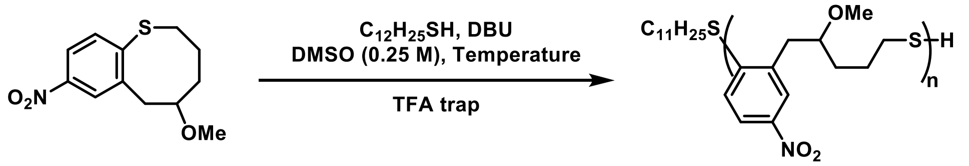

BT2: targeting DP = 25

entry	Temp. (°C)	Temp. (K)	1/T (K^{-1})	conversion (%)	$[M]_0$	$[M]_e$	In $[M]_e$
1	31 °C	304	0.003289	86.39%	0.25 M	0.0340 M	-3.3814
2	60 °C	333	0.003003	73.30%	0.25 M	0.06675 M	-2.7068
3	90 °C	363	0.002755	52.72%	0.25 M	0.1182 M	-2.1354
4	120 °C	393	0.002545	33.55%	0.25 M	0.1661 M	-1.7950

The thermodynamic parameters can be extracted by the linear fitting of the plot of $\ln[M]_e$ against *1/T* according to the following equation:

$$\mathrm{In}[M]_e = \frac{\Delta H}{RT} - \frac{\Delta S}{R}$$

Here $[M]_e$ is the monomer concentration at thermodynamic equilibrium in mol L^{-1}, T is the reaction temperature in K^{-1}, ΔH is the enthalpy change of polymerization in kJ mol^{-1}, ΔS is the entropy change of polymerization in J mol^{-1} K^{-1}, and R is the gas constant (8.314 J mol^{-1} K^{-1}).

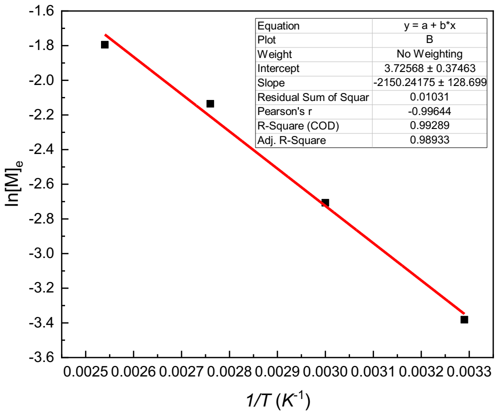

Figure S22. The van 't Hoff plot of **BT2**.

$$\Delta H = -2150 \text{ x } 8.314 \text{ J mol}^{-1} = -17.88 \text{ kJ mol}^{-1}$$

$$\Delta S = -3.73 \text{ x } 8.314 \text{ J mol}^{-1} \text{ K}^{-1} = -31.01 \text{ J mol}^{-1} \text{ K}^{-1}$$

$$T_c \text{ (0.25 M)} = -17880 \div (-31.01) \text{ K} = 576.6 \text{ K}$$

11. X-ray crystal structure of the monomers BT1 and BT2

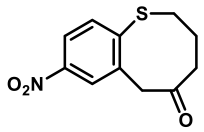

BT1

≡

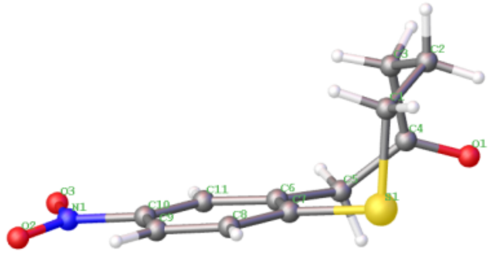

CCDC: 2219284

Experimental. Single crystals of $C_{11}H_{11}NO_3S$ **BT1** were prepared by slow evaporation of hexane/ethyl acetate solution. Single colorless needle-shaped crystals of **BT1** were chosen from the sample as supplied. A suitable crystal with dimensions 0.60 × 0.42 × 0.18 mm^3 was selected and mounted on a loop with paratone on a XtaLAB Synergy-S diffractometer. The crystal was kept at a steady T = 105(7) K during data collection. The structure was solved with the **ShelXT** 2018/2 (Sheldrick, 2018) solution program using dual methods and by using **Olex2** 1.5-alpha (Dolomanov et al., 2009) as the graphical interface. The model was refined with **ShelXL** 2018/3 (Sheldrick, 2015) using full matrix least squares minimisation on F^2.

Crystal Data. $C_{11}H_{11}NO_3S$, M_r = 237.27, triclinic, P-1 (No. 2), a = 7.0144(2) Å, b = 7.4462(2) Å, c = 10.7524(3) Å, α = 77.694(2)°, β = 82.884(2)°, γ = 72.182(2)°, V = 521.34(3) Å^3, T = 105(7) K, Z = 2, Z' = 1, μ (Mo K$_\alpha$) = 0.300, 26396 reflections measured, 5406 unique (R_{int} = 0.0409) which were used in all calculations. The final wR_2 was 0.0874 (all data) and R_1 was 0.0298 (I≥2 σ(I)).

Table S7: Crystallographic data and structure refinement for **BT1**.

**Compound**	**BT1**
Formula	$C_{11}H_{11}NO_3S$
$D_{calc.}$/ g cm^{-3}	1.511
μ/mm^{-1}	0.300
Formula Weight	237.27
Color	colorless
Shape	prism-shaped
Size/mm^3	0.60×0.42×0.18
T/K	105(7)
Crystal System	triclinic
Space Group	*P*-1
a/Å	7.0144(2)
b/Å	7.4462(2)
c/Å	10.7524(3)
α/°	77.694(2)
β/°	82.884(2)
γ/°	72.182(2)
V/Å^3	521.34(3)
Z	2
Z'	1
Wavelength/Å	0.71073
Radiation type	Mo K_{α}
Θ_{min}/°	1.942
Θ_{max}/°	37.982
Measured Refl's.	26396
Indep't Refl's	5406
Refl's I≥2 σ(I)	4945
R_{int}	0.0409
Parameters	259
Restraints	270
Largest Peak	0.527
Deepest Hole	-0.273
GooF	1.041
wR_2 (all data)	0.0874
wR_2	0.0862
R_1 (all data)	0.0325
R_1	0.0298

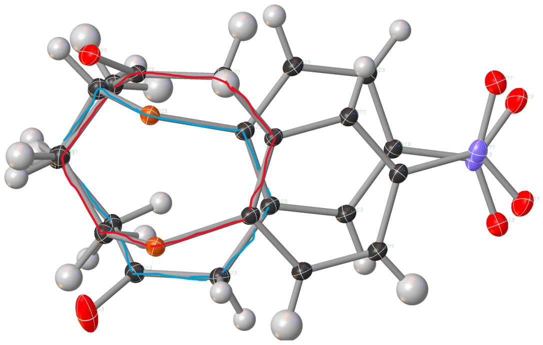

Figure S13: The disordered structure of **BT1**. There are two components, each rotated about the atom C2 with populations of 0.977 and 0.023.

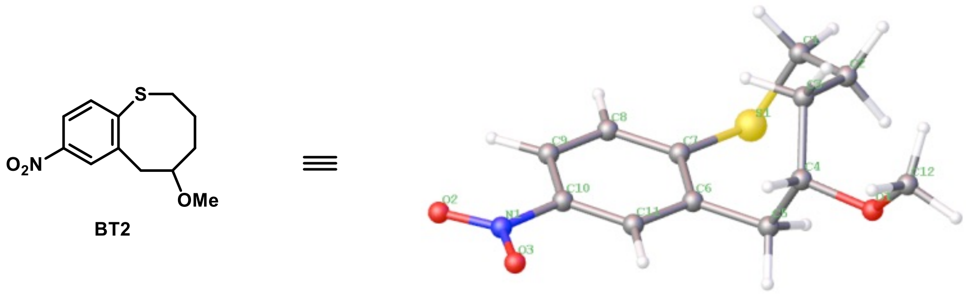

CCDC: 2219282

Experimental. Single crystals of $C_{12}H_{15}NO_3S$ **BT2** were prepared by slow evaporation of hexane/ethyl acetate solution. Single colorless needle-shaped crystals of **BT2** were chosen from the sample as supplied. A suitable crystal with dimensions 0.33 × 0.25 × 0.12 mm^3 was selected and mounted on a loop with paratone on a XtaLAB Synergy-S diffractometer. The crystal was kept at a steady T = 99.9(5) K during data collection. The structure was solved with the **ShelXT** 2018/2 (Sheldrick, 2018) solution program using dual methods and by using **Olex2** 1.5-alpha (Dolomanov et al., 2009) as the graphical interface. The model was refined with **olex2.refine** 1,5-alpha (Bourhis et al., 2015) using full matrix least squares minimisation on $\boldsymbol{F^2}$.

Crystal Data. $C_{12}H_{15}NO_3S$, M_r = 253.324, monoclinic, $P2_1/c$ (No. 14), a = 7.1742(3) Å, b = 20.6794(8) Å, c = 8.7098(4) Å, β = 110.751(5)°, $\alpha = \gamma$ = 90°, V = 1208.34(10) Å^3, T = 99.9(5) K, Z = 4, Z' = 1, μ (Cu K_α) = 2.365, 13258 reflections measured, 2299 unique (R_{int} = 0.0450) which were used in all calculations. The final wR_2 was 0.0474 (all data) and R_1 was 0.0201 (I≥2 σ(I)).

Table S8: Crystallographic data and structure refinement for **BT2**.

**Compound**	**BT2**
Formula	$C_{12}H_{15}NO_3S$
$D_{calc.}$/ g cm^{-3}	1.393
μ/mm^{-1}	2.365
Formula Weight	253.324
Color	colorless
Shape	needle-shaped
Size/mm^3	0.33×0.25×0.12
T/K	99.9(5)
Crystal System	monoclinic
Space Group	$P2_1/c$
a/Å	7.1742(3)
b/Å	20.6794(8)
c/Å	8.7098(4)
α/°	90
β/°	110.751(5)
γ/°	90
V/Å^3	1208.34(10)
Z	4
Z'	1
Wavelength/Å	1.54184
Radiation type	Cu K_{α}
Θ_{min}/°	4.28
Θ_{max}/°	72.89
Measured Refl's.	13258
Indep't Refl's	2299
Refl's I≥2 σ(I)	2182
R_{int}	0.0450
Parameters	290
Restraints	48
Largest Peak	0.2088
Deepest Hole	-0.1775
GooF	1.1198
wR_2 (all data)	0.0474
wR_2	0.0457
R_1 (all data)	0.0219
R_1	0.0201

12. Calculations of ring-opening enthalpy ΔH

The ring-opening enthalpy ΔH of these monomers was computed at the level of Density Functional Theory (DFT)[2] using the computational scheme that was described and demonstrated in our previous work.[3-4] In short, the procedure involves some steps. First, a monomer model and several polymer models, each of them is a closed loop created by a given number n of monomers (n

ranges from 3 to 6 in this work), are constructed using Polymer Structure Predictor package.[5] Then, the configuration space associated with each of these models is extensively explored at two separated levels using a combination of long classical molecular dynamics (MD) simulations and DFT-based MD simulations using VASP.[6] Next, the ring-opening enthalpy ΔH associated with the finite-size (n) polymer models is computed from the trajectories of the DFT-based MD simulation. Finally, ΔH (computed for the finite values of n) is extrapolated to the limit of infinite size of the polymer models, i.e., $n \rightarrow \infty$. The significant complexity of this procedure, which is also computational demanding, is required to reach the predictive capability on a wide variety of chemistries considered in this project.

13. NMR spectrum of products

^{1}H NMR (700 MHz, $CDCl_3$) and ^{13}C NMR (176 MHz, $CDCl_3$) spectra for BT1

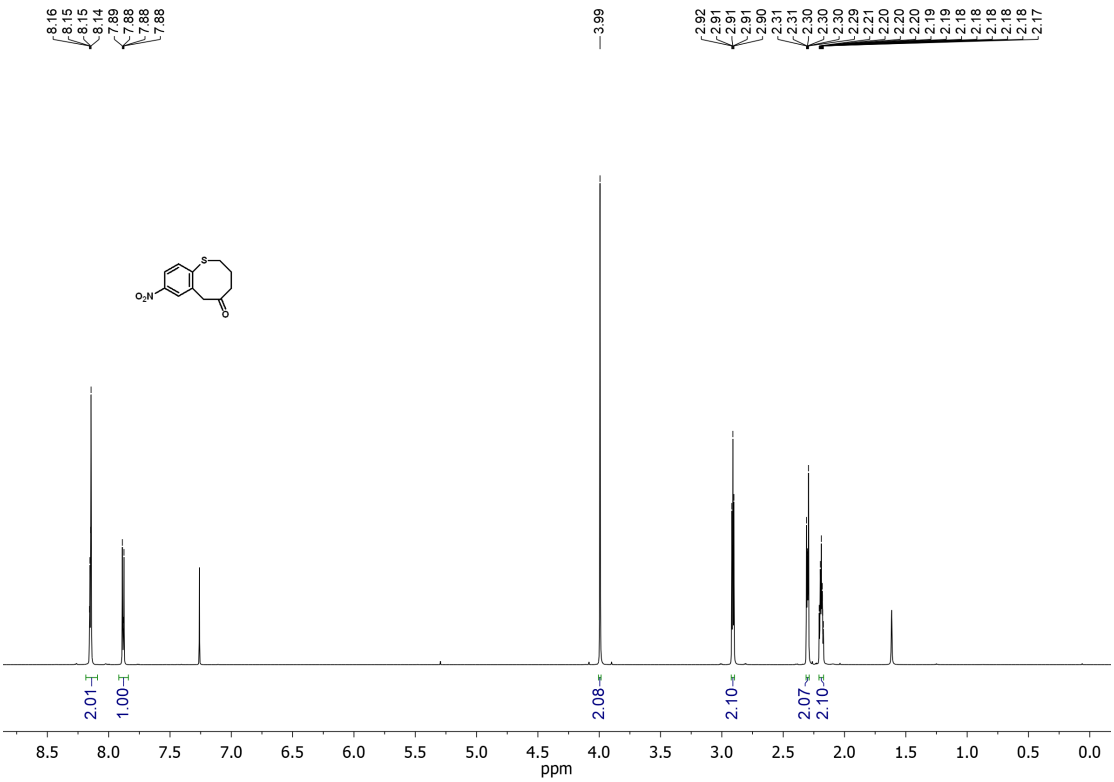

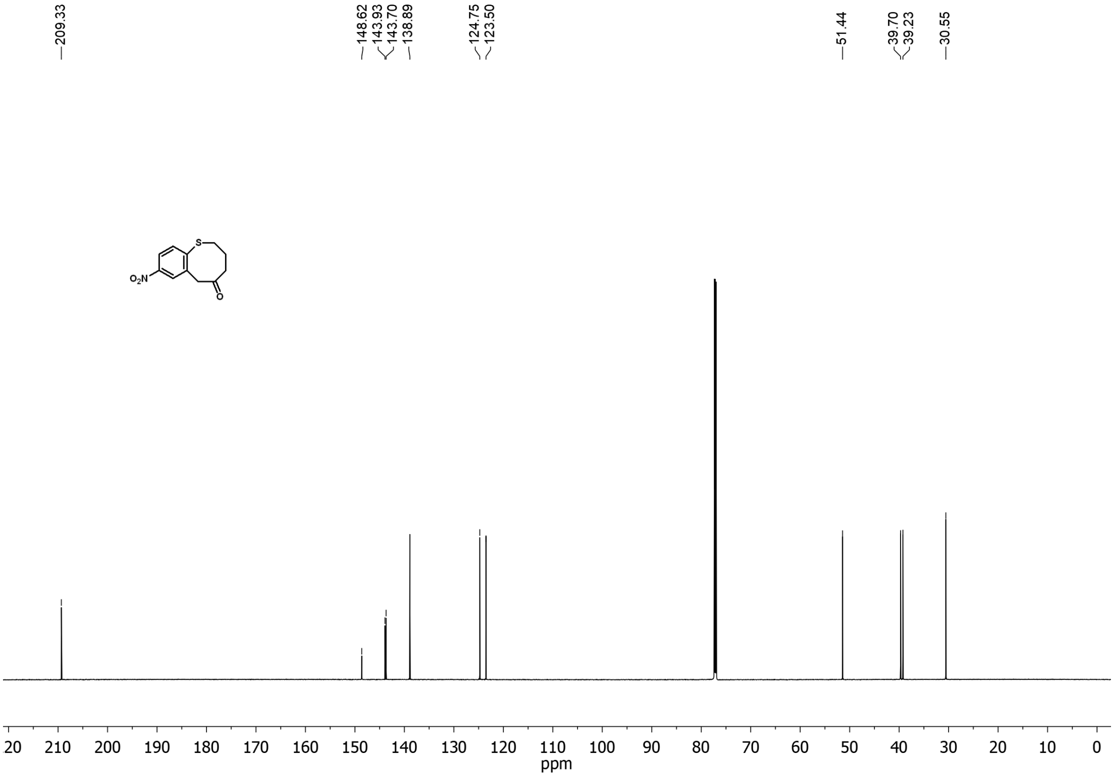

^{1}H NMR (700 MHz, $CDCl_3$) and ^{13}C NMR (176 MHz, $CDCl_3$) spectra for BT-OH

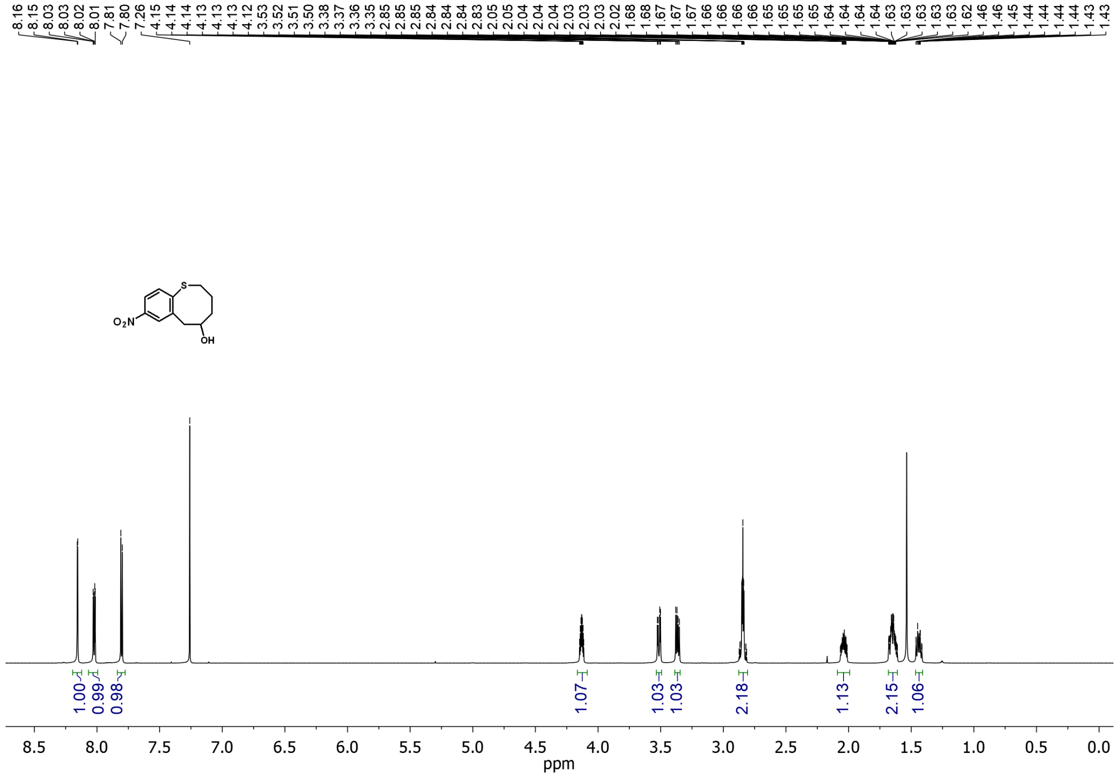

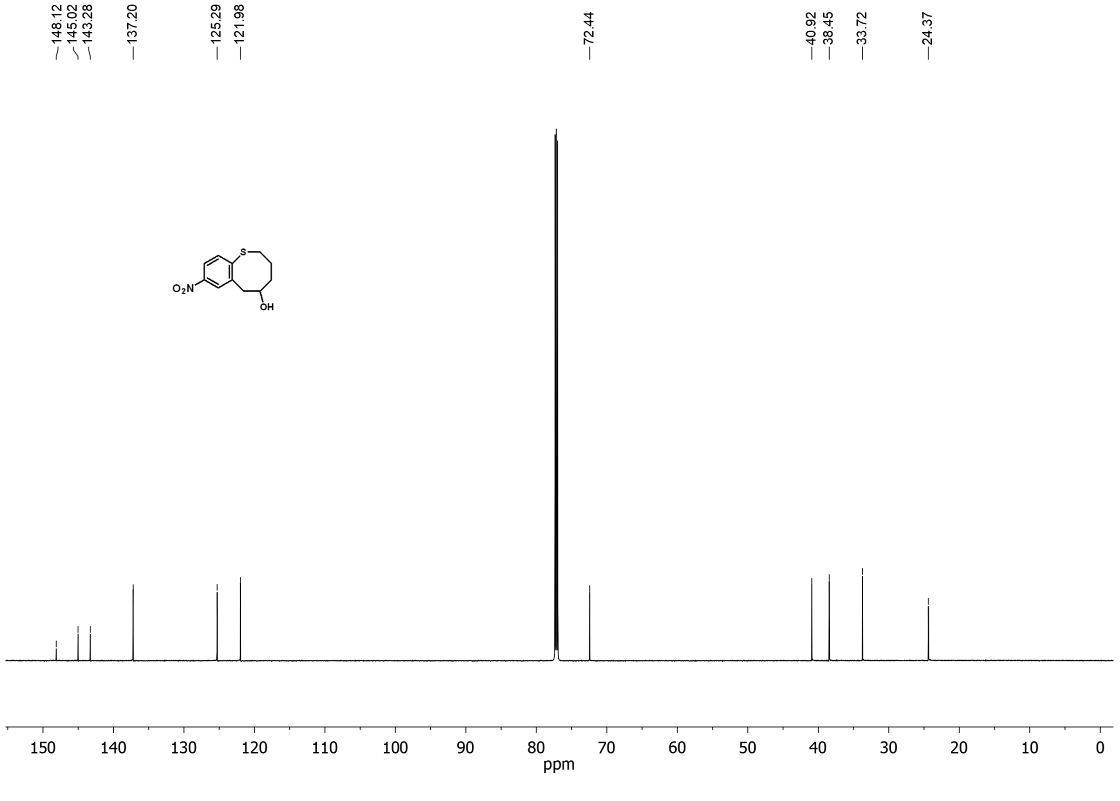

^{1}H NMR (700 MHz, $CDCl_3$) and ^{13}C NMR (176 MHz, $CDCl_3$) spectra for BT2

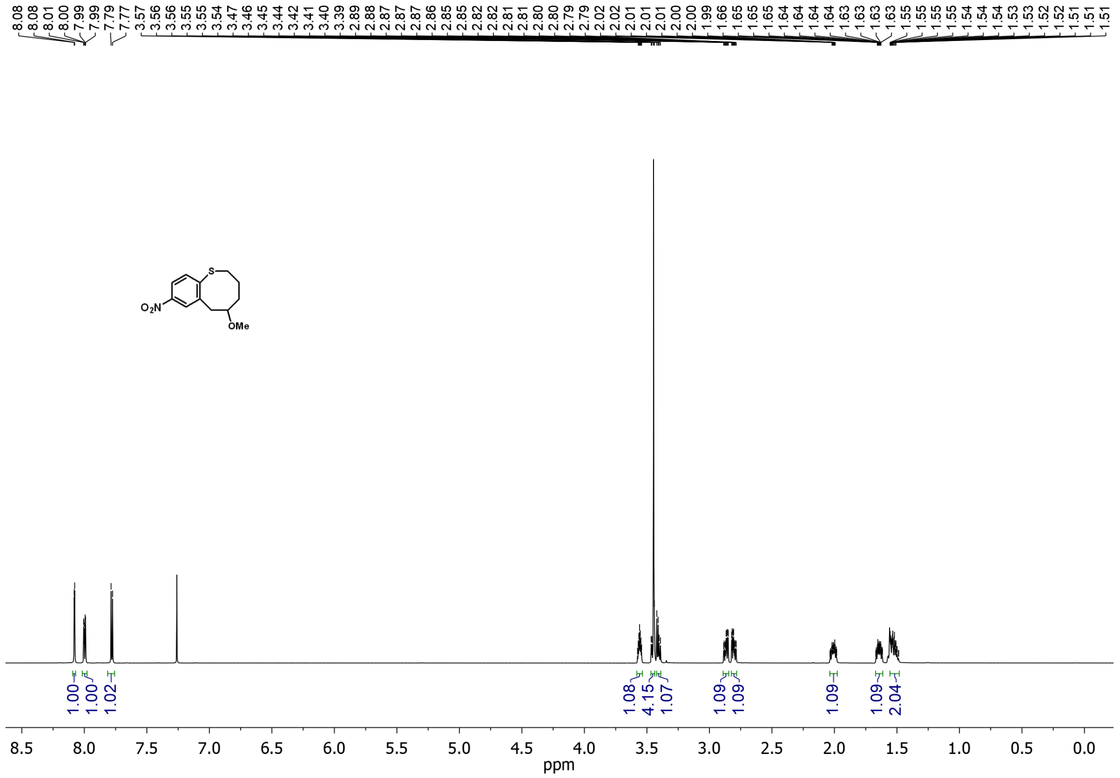

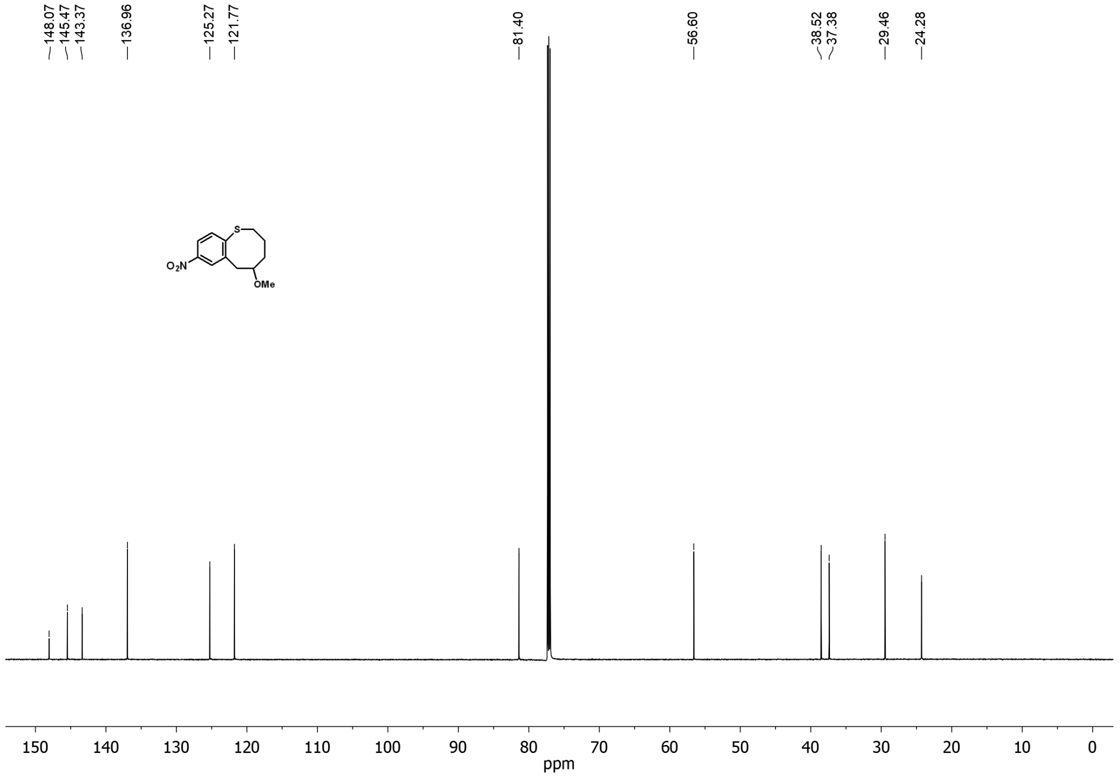

^{1}H NMR (700 MHz, $CDCl_3$) and ^{13}C NMR (176 MHz, $CDCl_3$) spectra for BT3

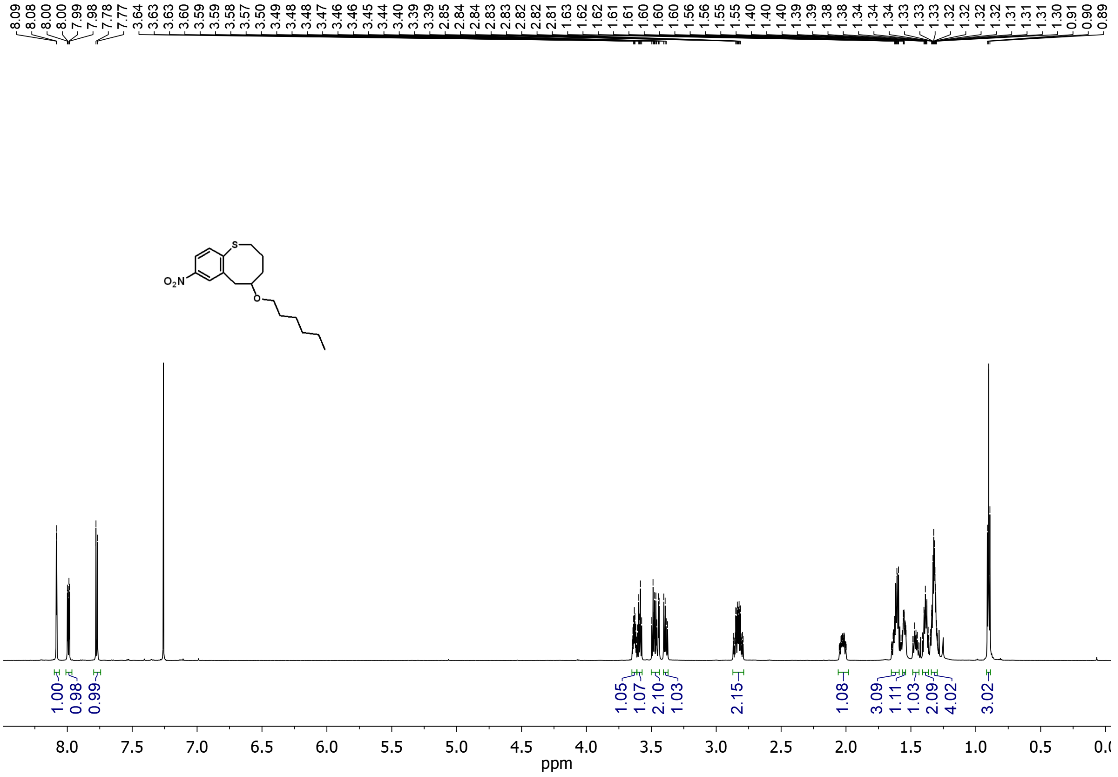

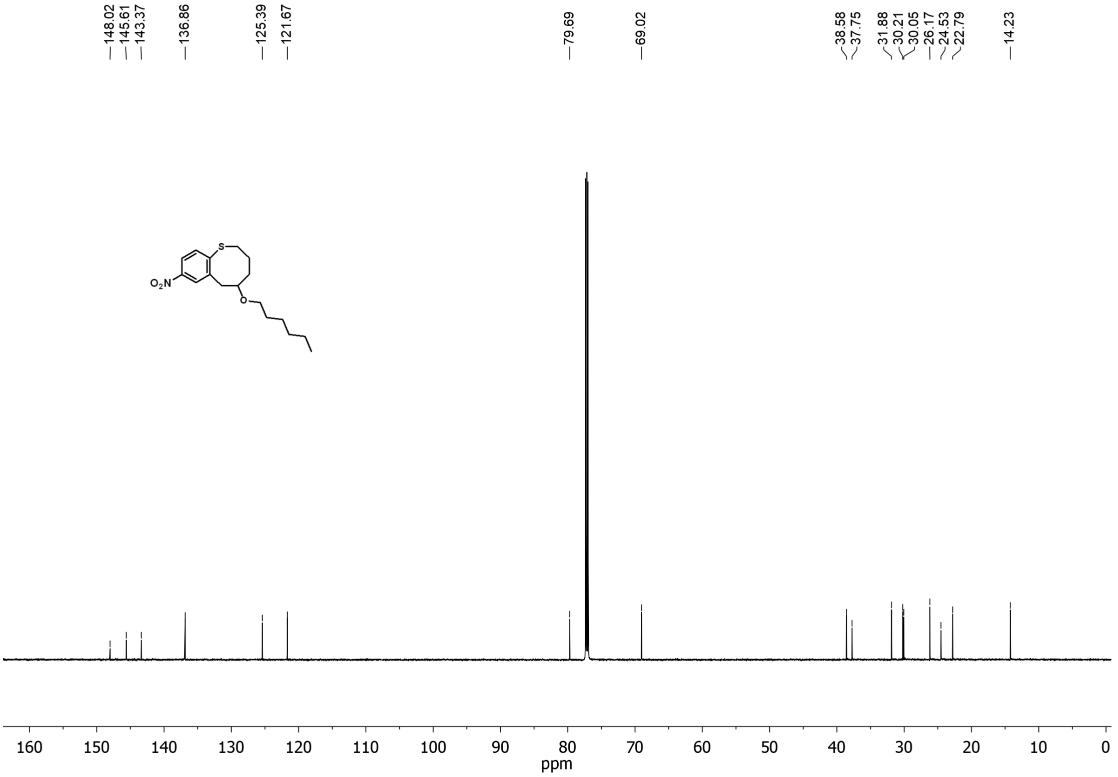

^{1}H NMR (700 MHz, CDCl3) and ^{13}C NMR (176 MHz, CDCl3) spectra for BT4

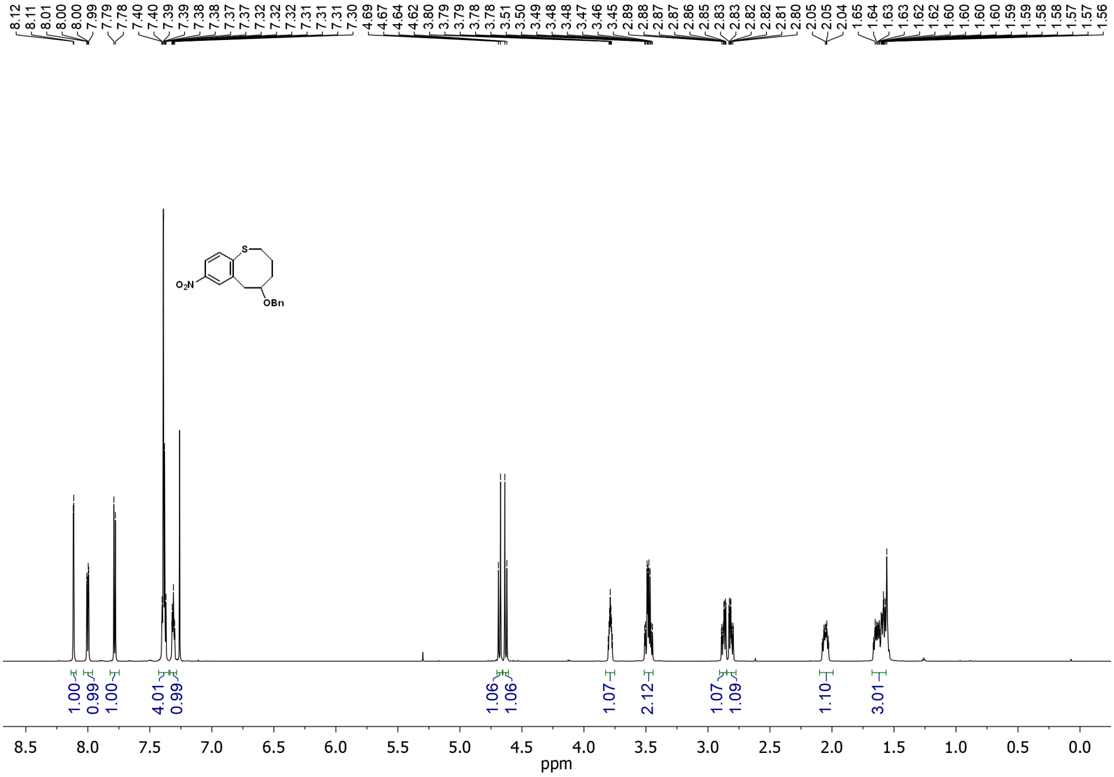

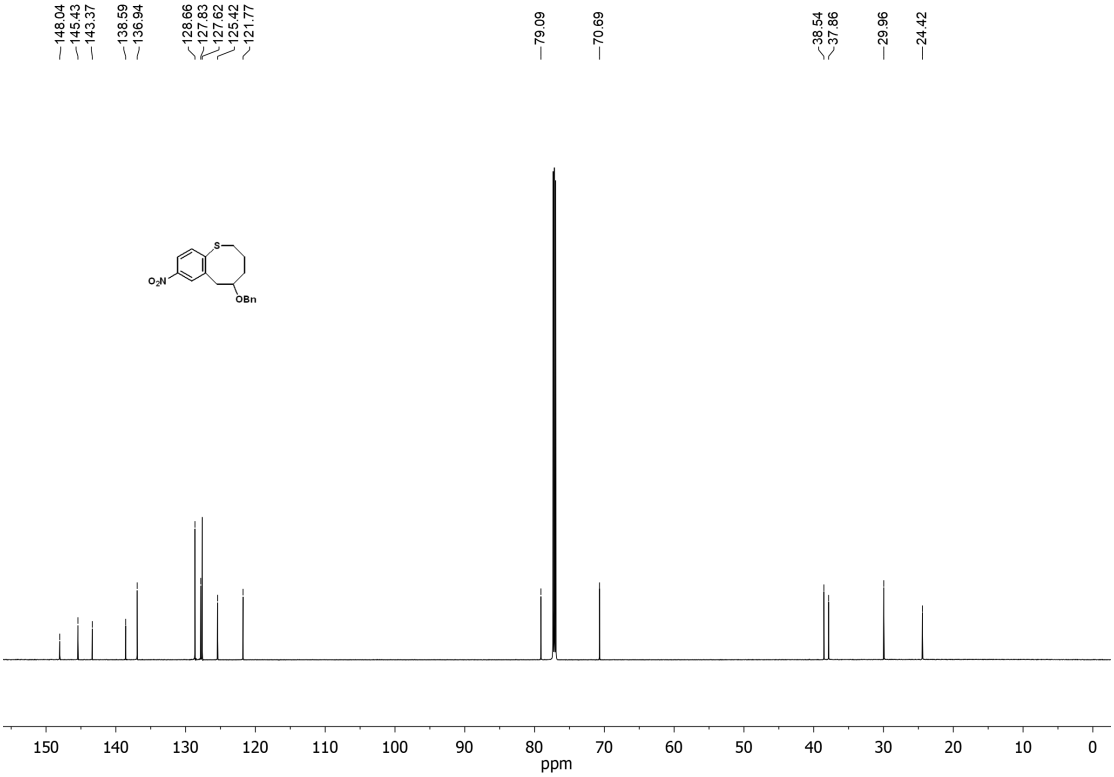

^{1}H NMR (700 MHz, $CDCl_3$) and ^{13}C NMR (176 MHz, $CDCl_3$) spectra for BT5

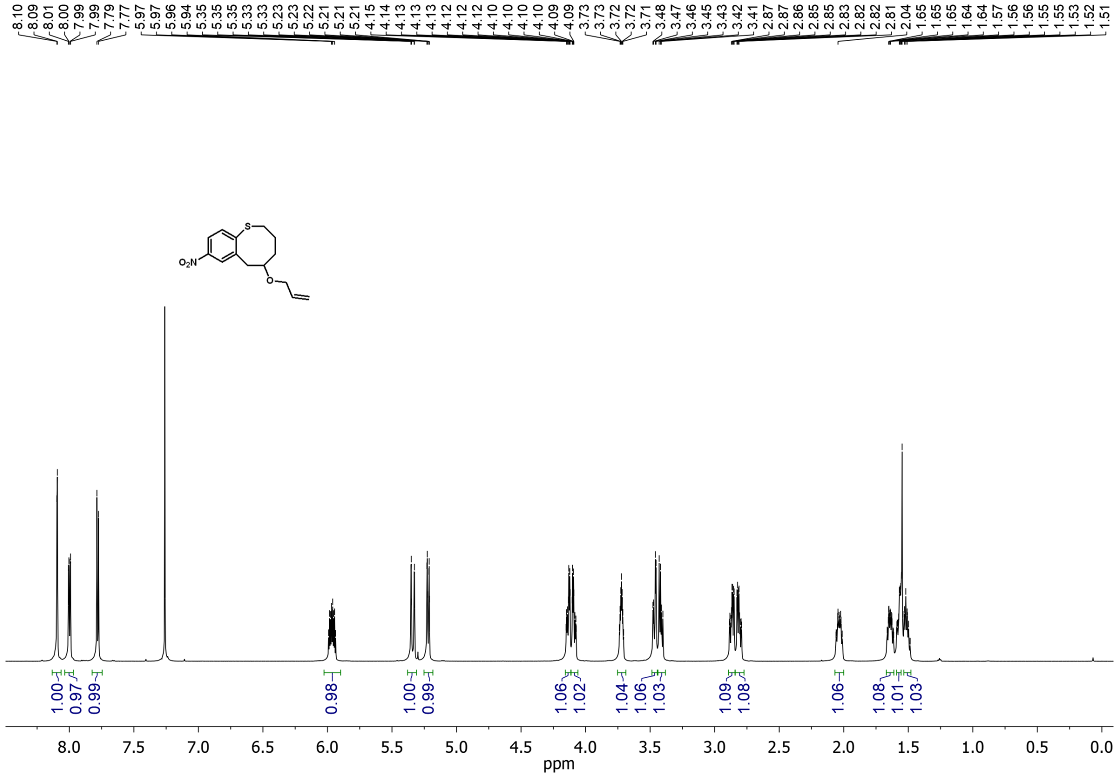

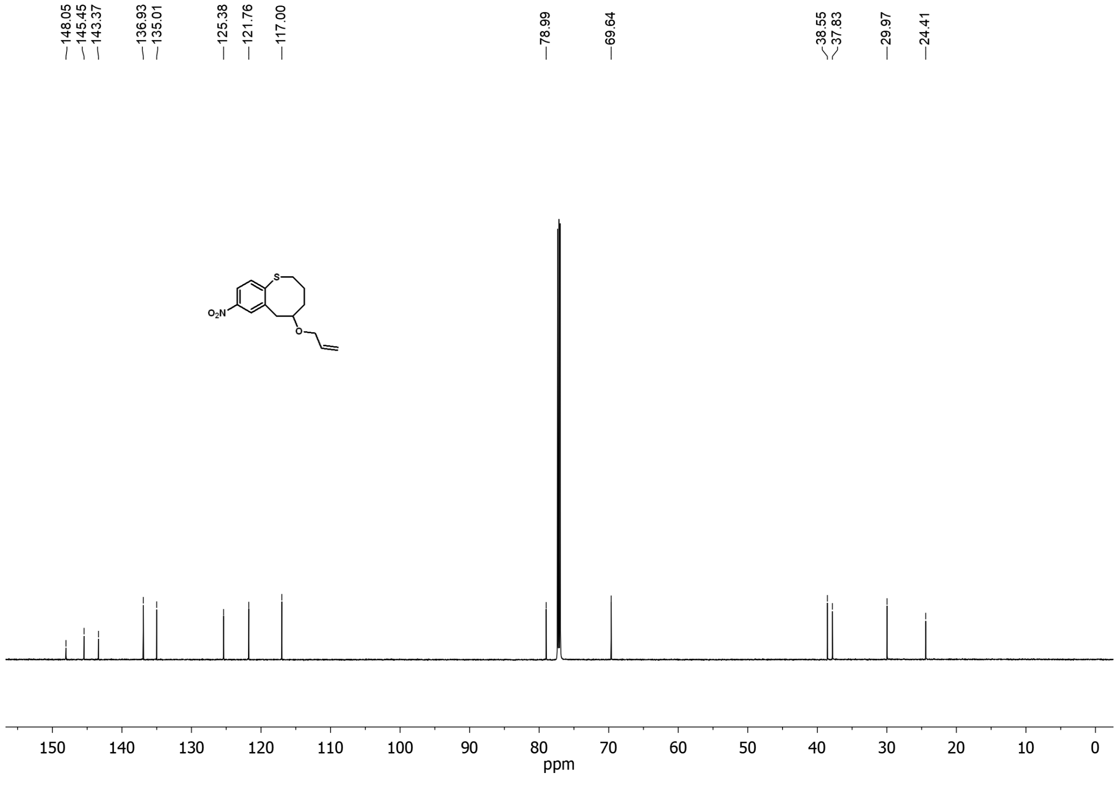

^{1}H NMR (700 MHz, CDCl3) and ^{13}C NMR (176 MHz, CDCl3) spectra for BT6

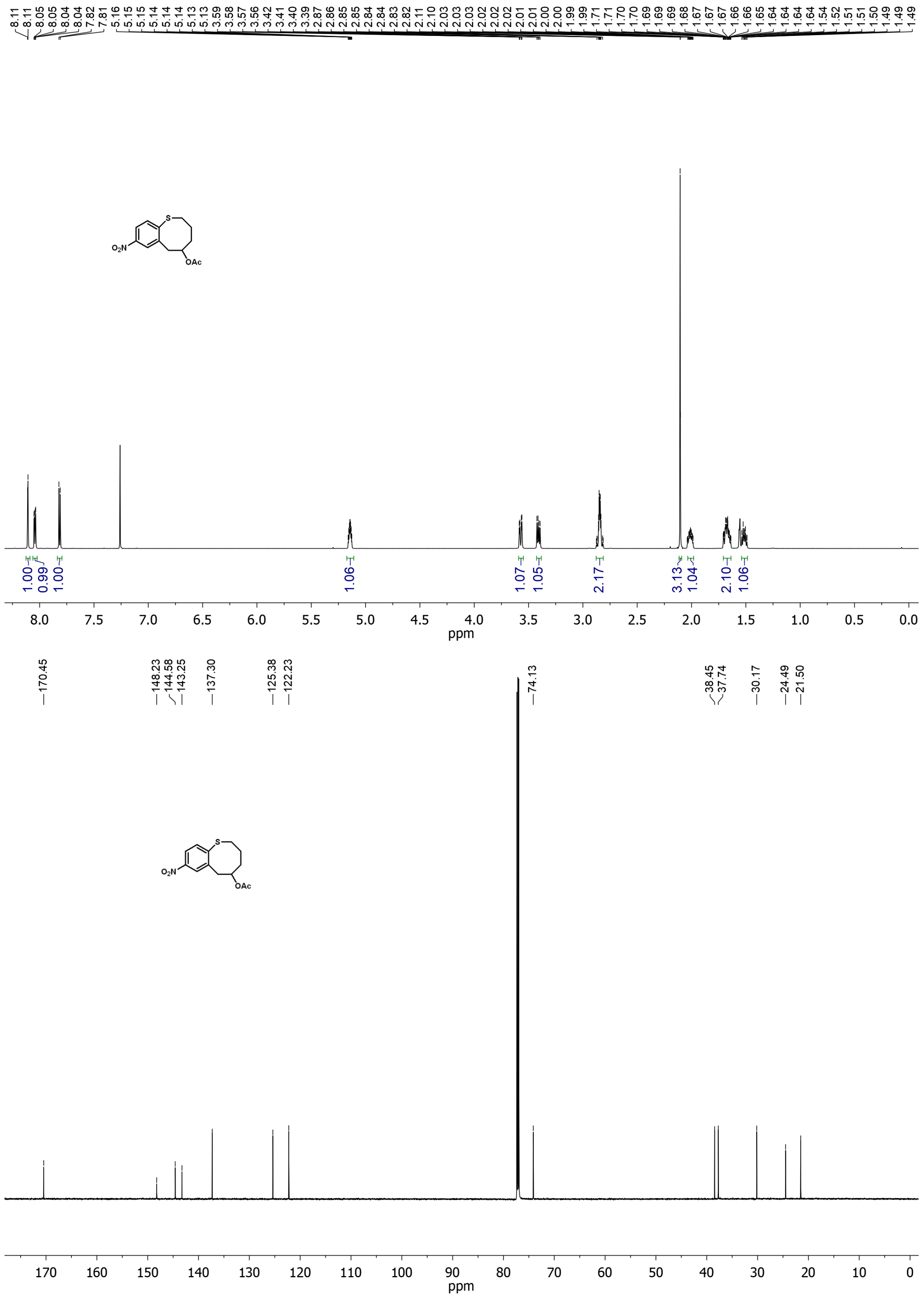

^{1}H NMR (700 MHz, $CDCl_3$) and ^{13}C NMR (176 MHz, $CDCl_3$) spectra for BT7

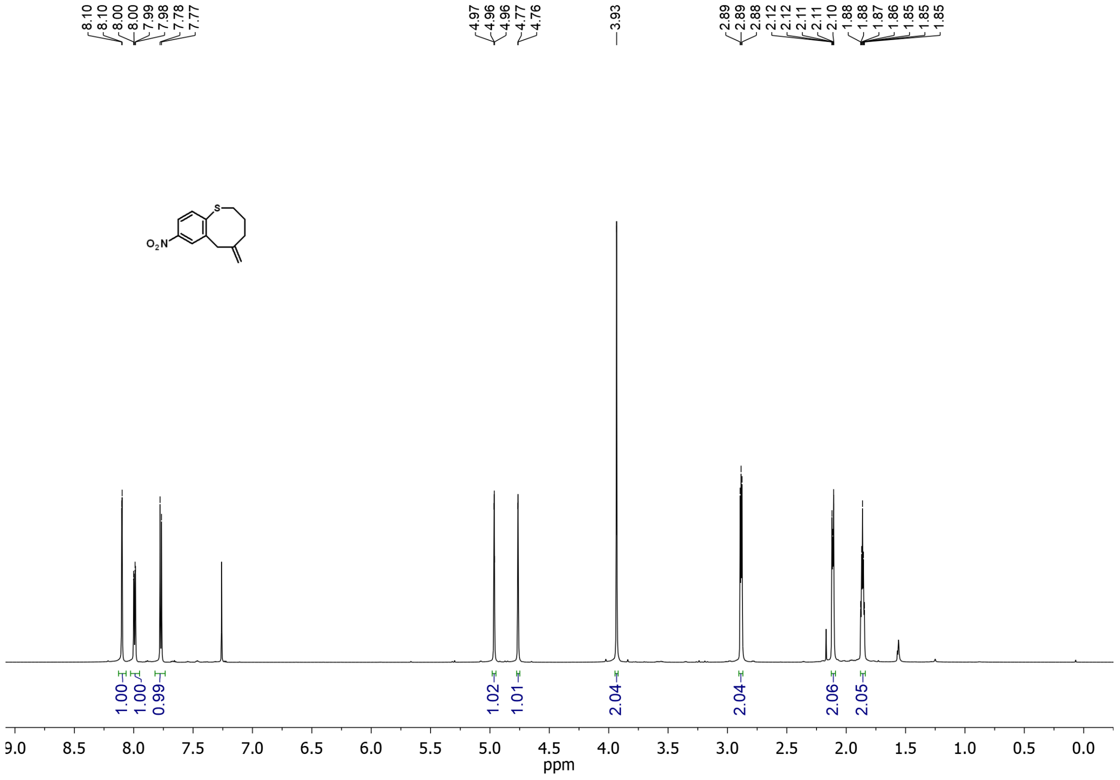

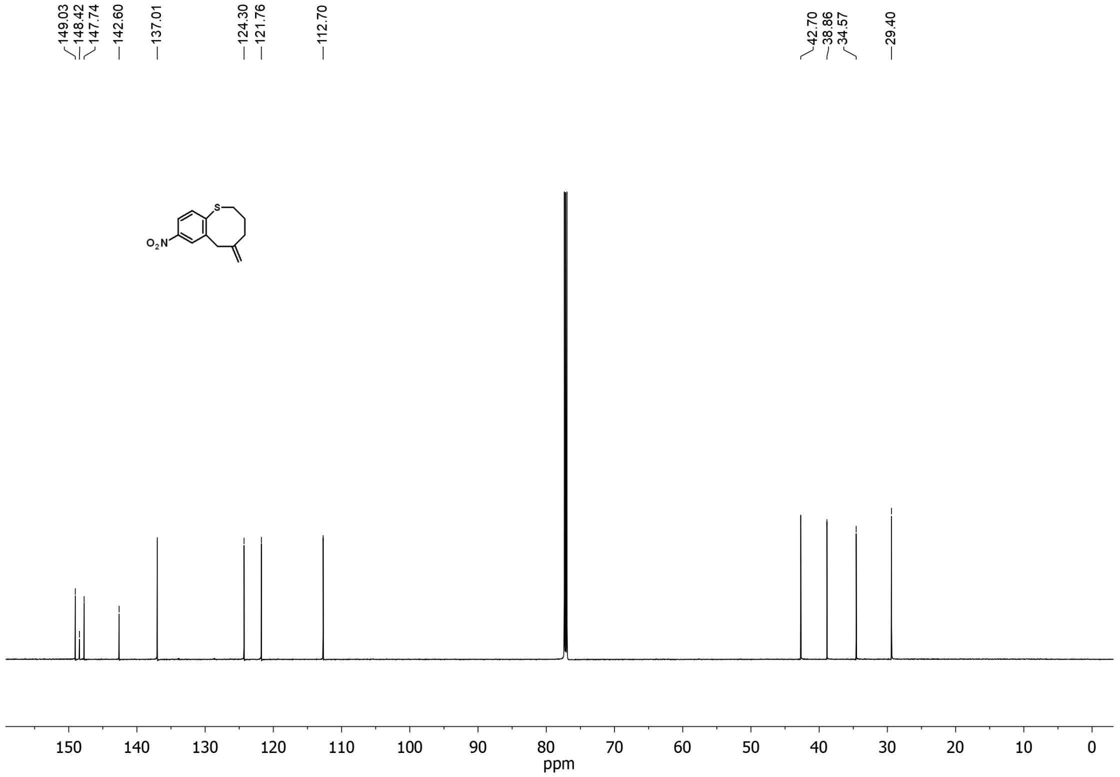

^{1}H NMR (700 MHz, CDCl3) and ^{13}C NMR (176 MHz, CDCl3) spectra for PBT2

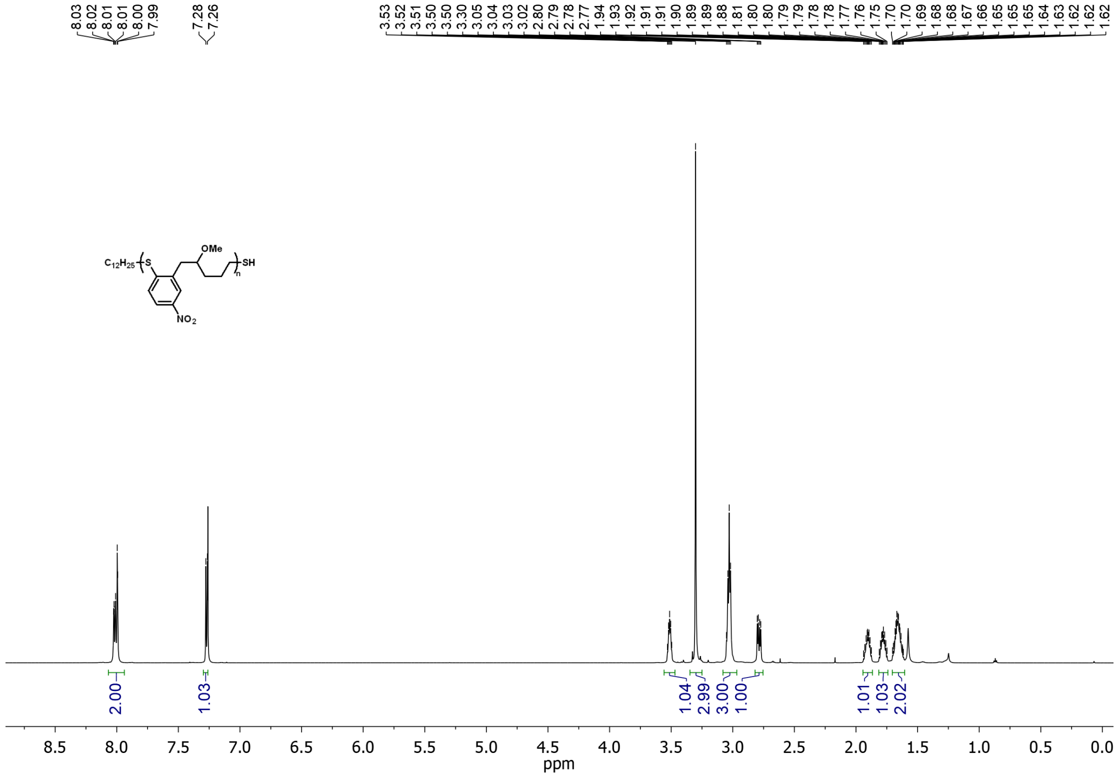

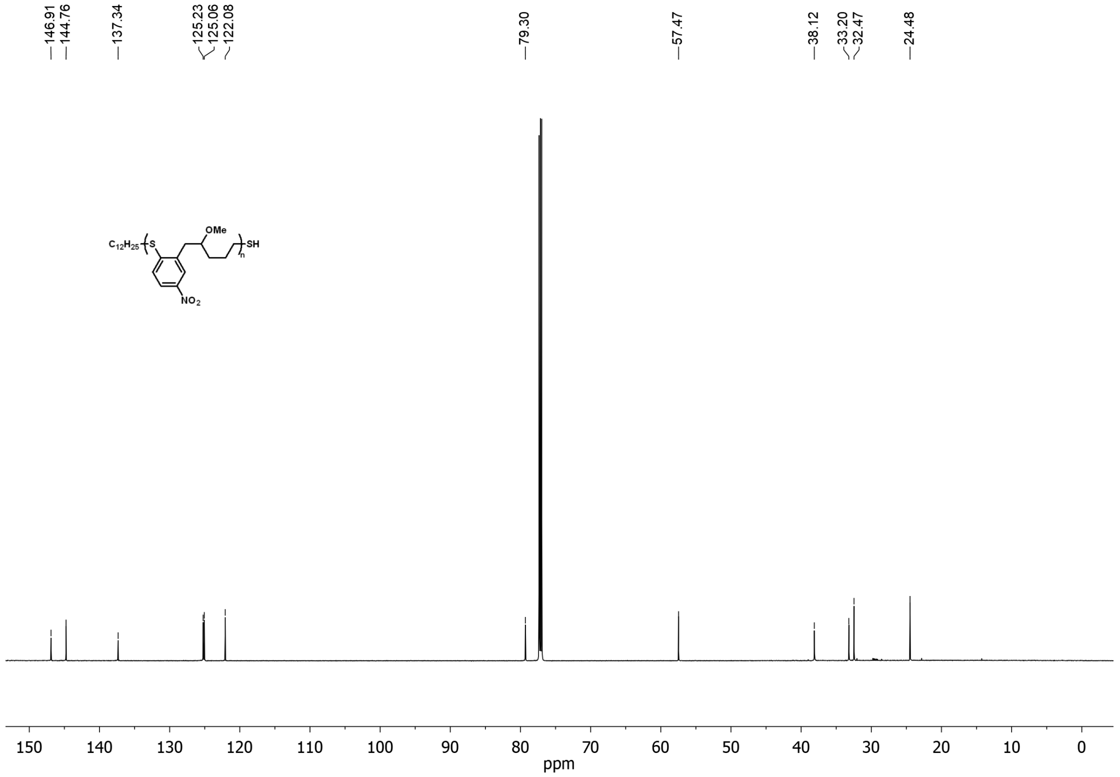

^{1}H NMR (700 MHz, $CDCl_3$) and ^{13}C NMR (176 MHz, $CDCl_3$) spectra for PBT3

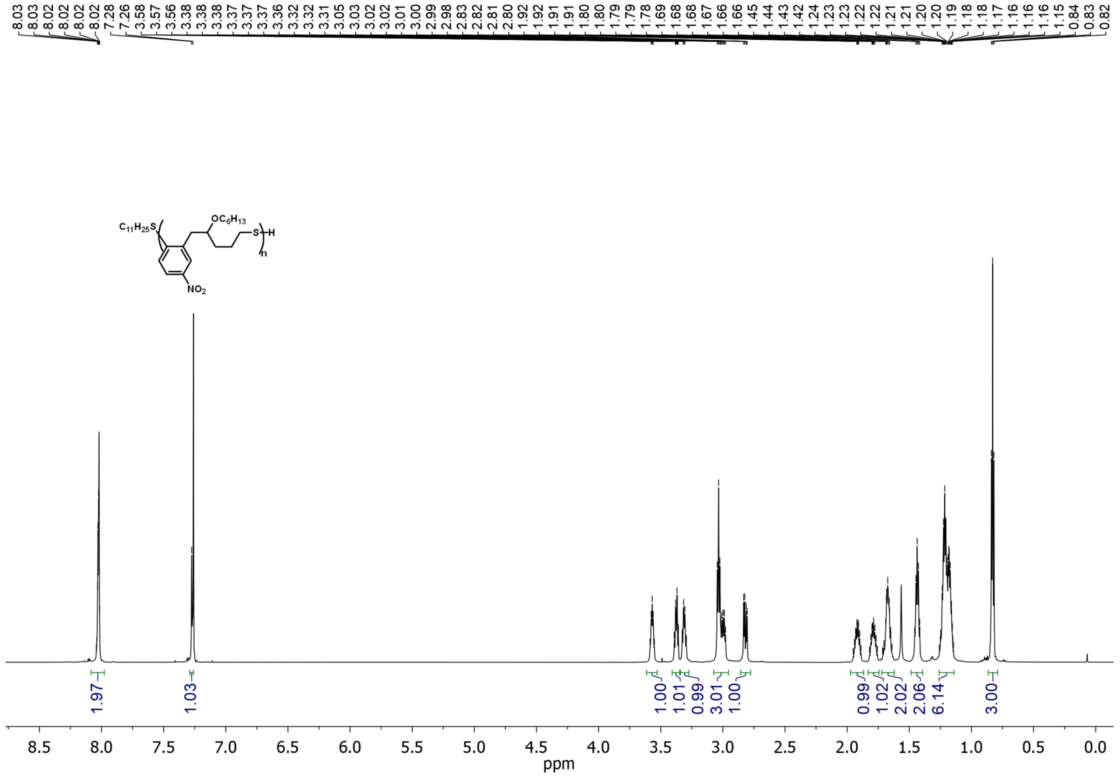

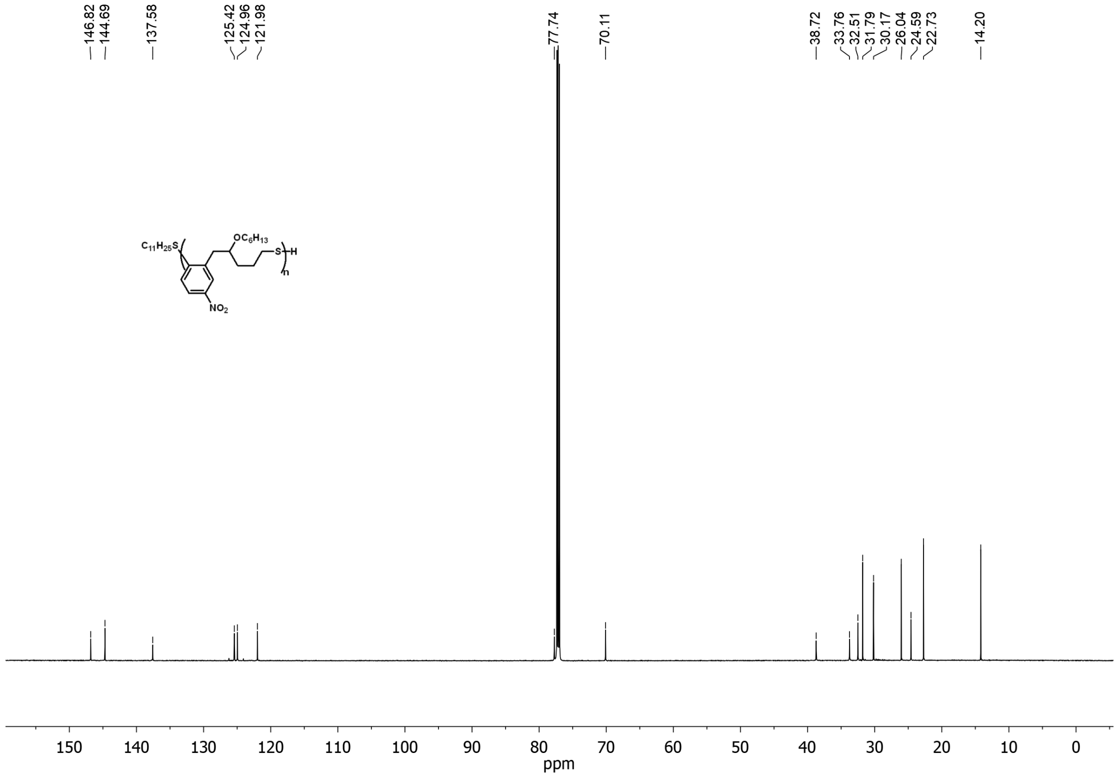

^{1}H NMR (700 MHz, CDCl3) and ^{13}C NMR (176 MHz, CDCl3) spectra for PBT4

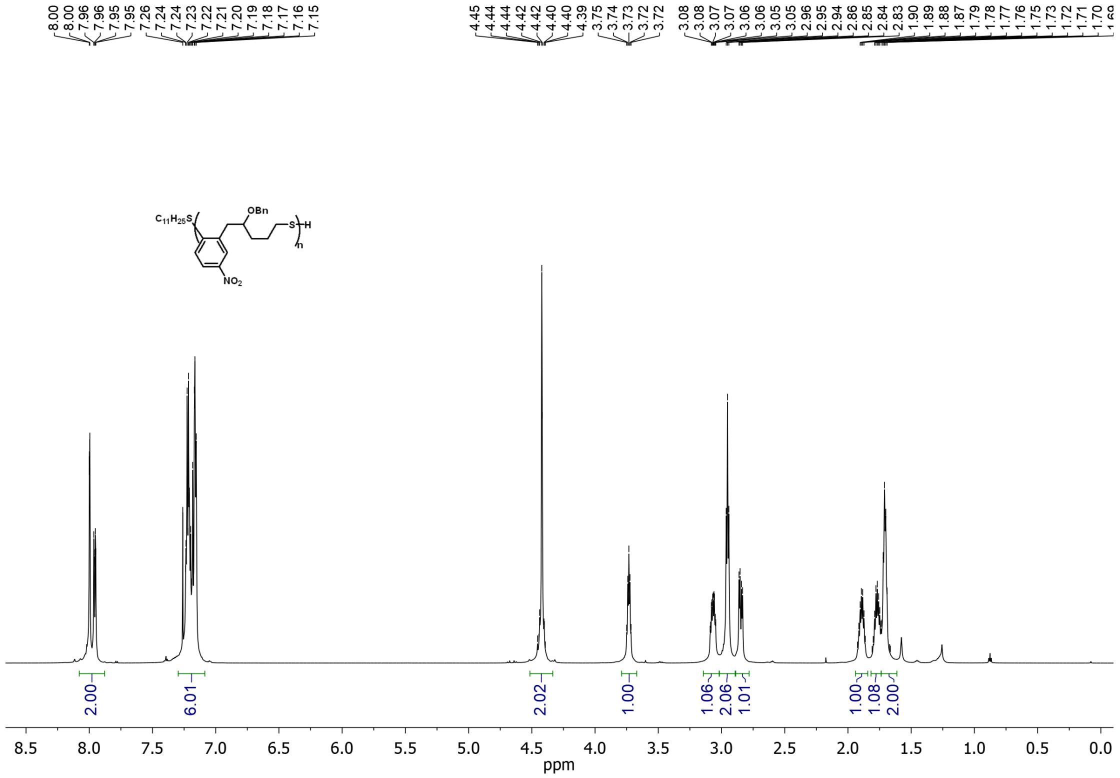

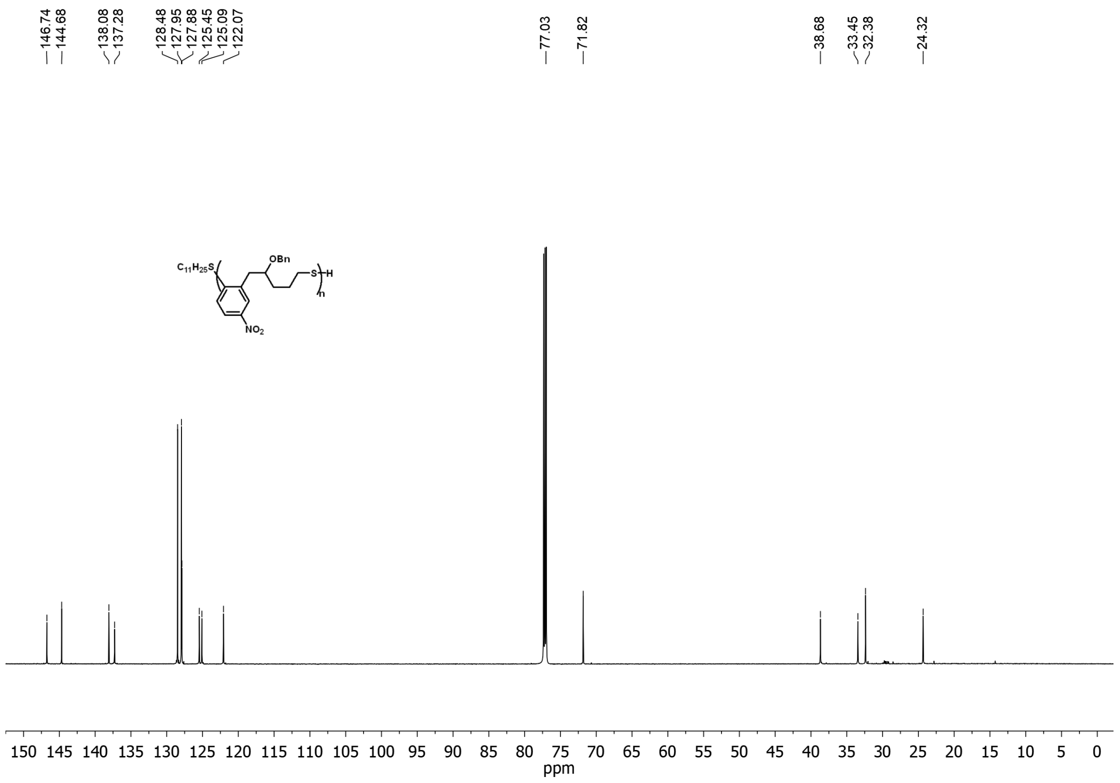

^{1}H NMR (700 MHz, $CDCl_3$) and ^{13}C NMR (176 MHz, $CDCl_3$) spectra for PBT5

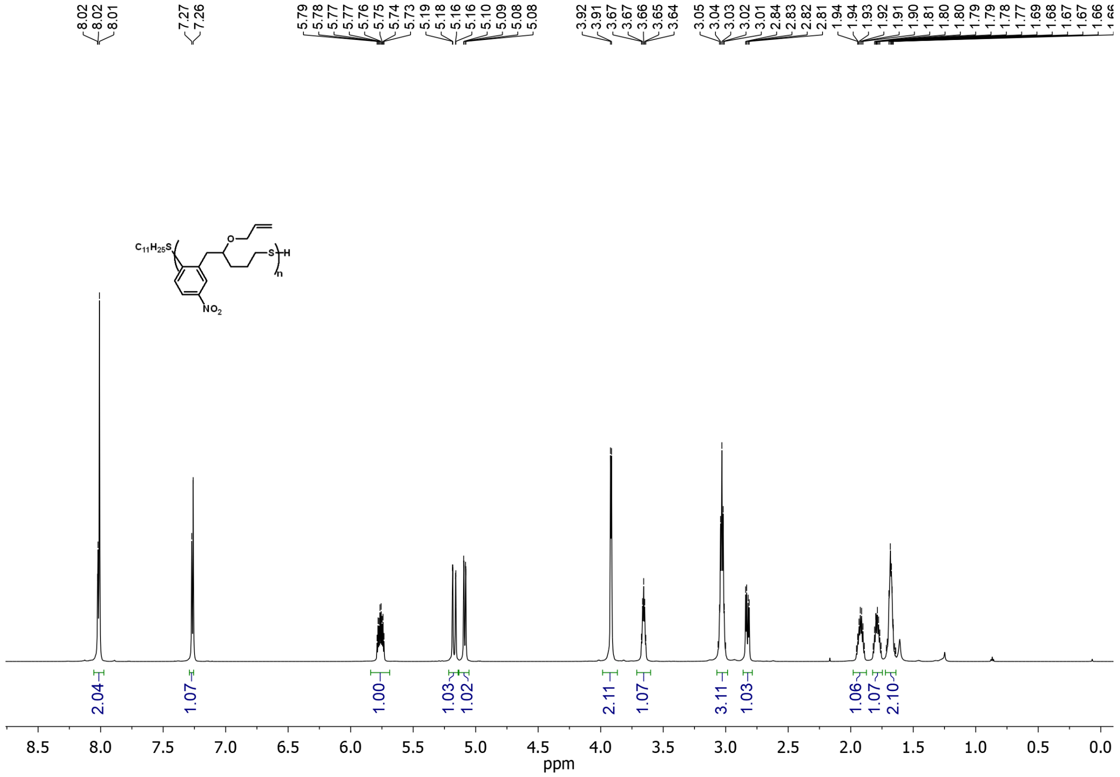

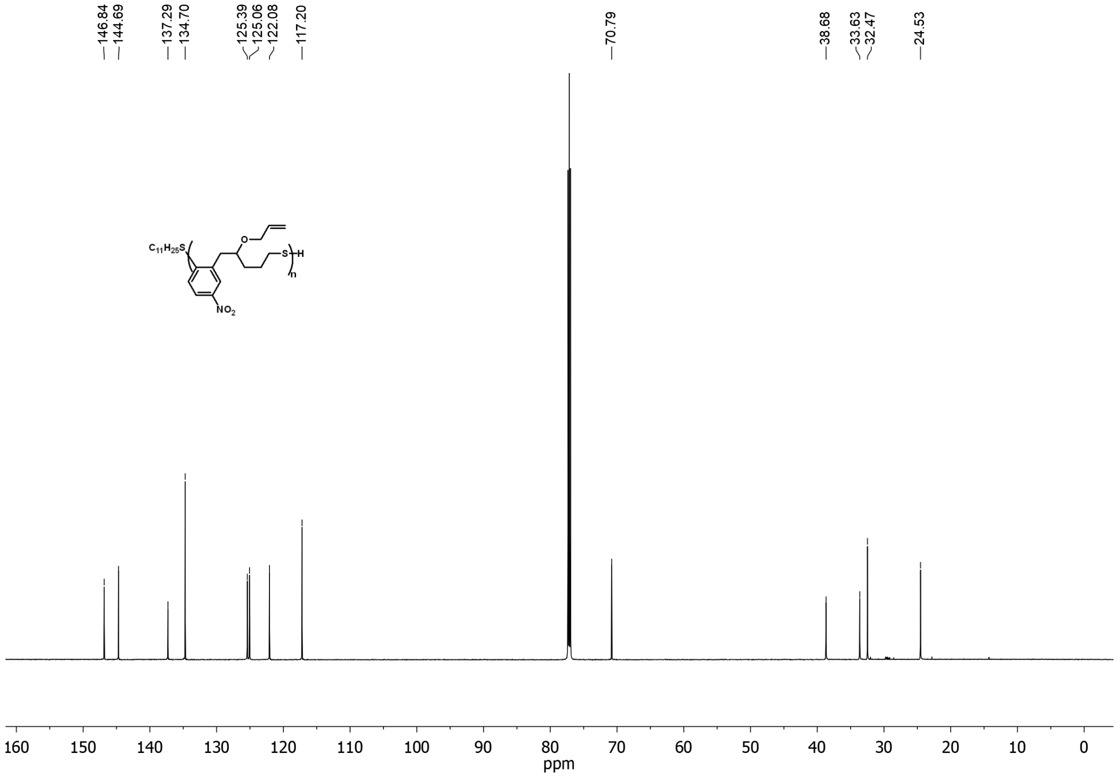

^{1}H NMR (700 MHz, $CDCl_3$) and ^{13}C NMR (176 MHz, $CDCl_3$) spectra for PBT6

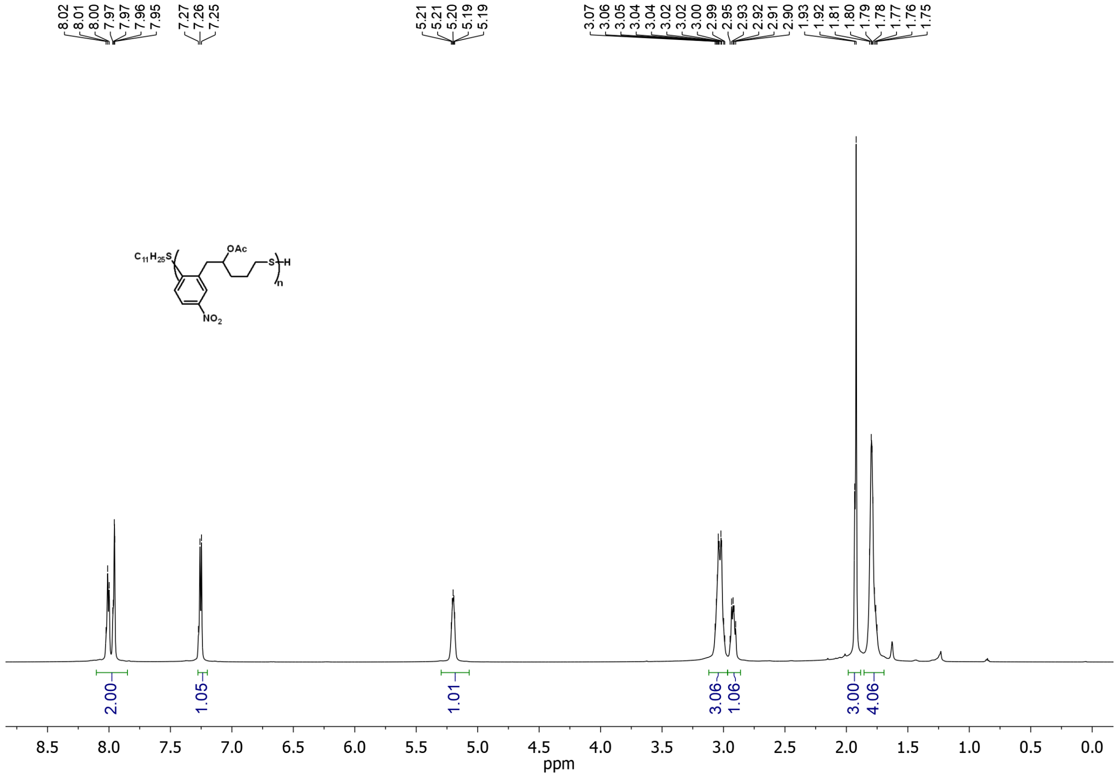

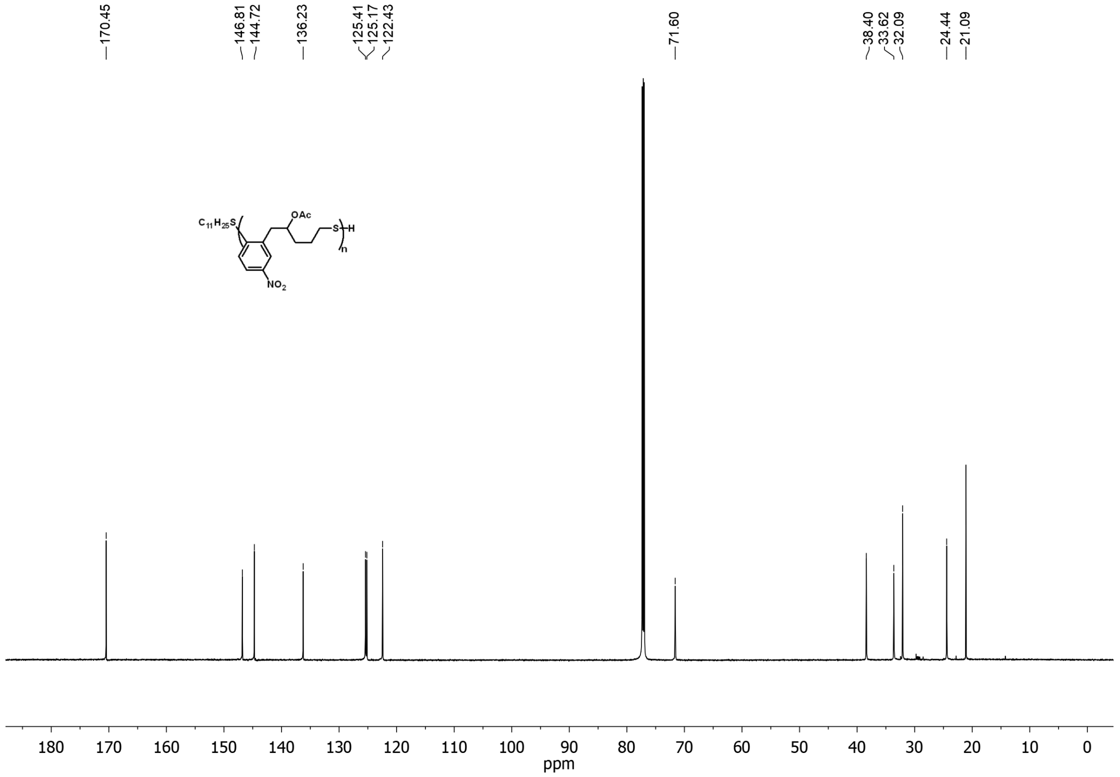

^{1}H NMR (700 MHz, $CDCl_3$) and ^{13}C NMR (176 MHz, $CDCl_3$) spectra for PBT7

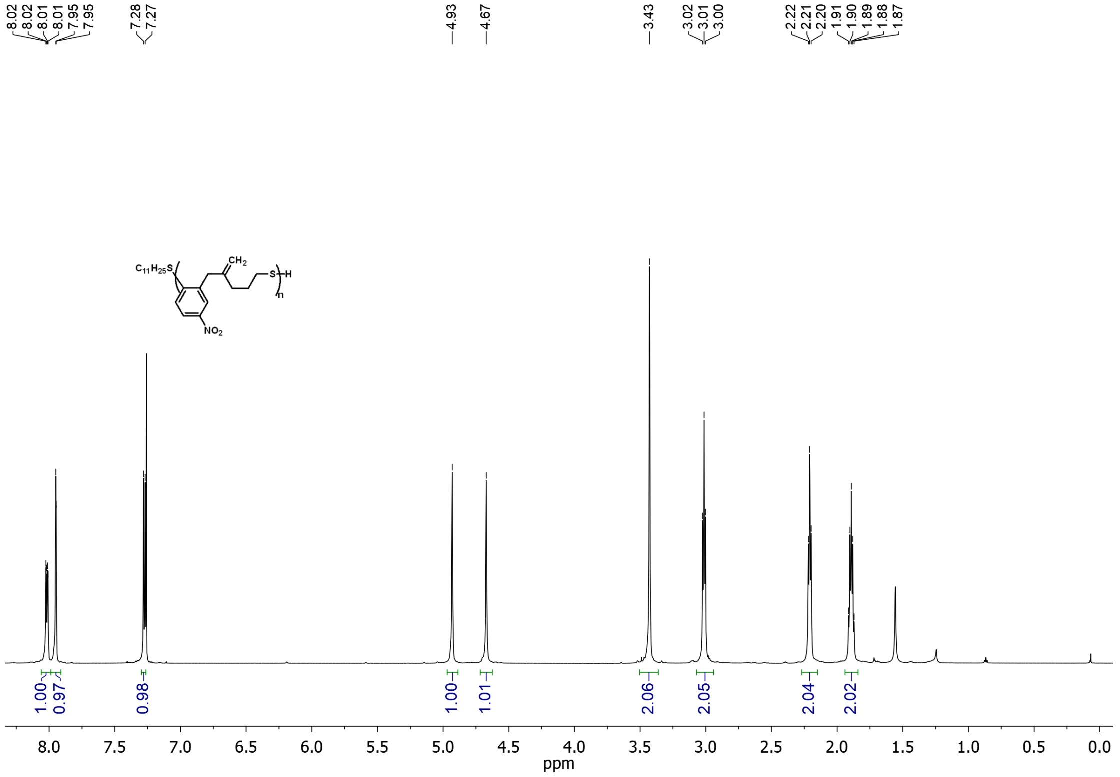

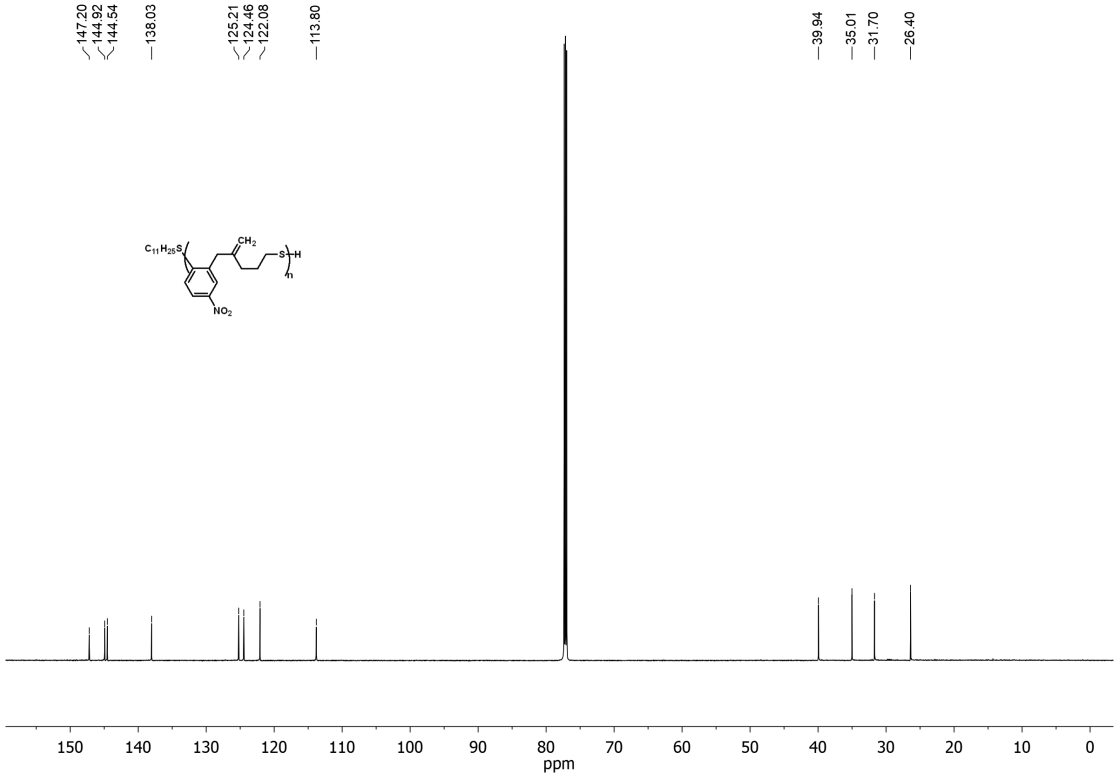

14. References

1. Lu, B.; Li, Y.; Wang, Y.; Aue, D. H.; Luo, Y.; Zhang, L. [3,3]- Sigmatropic Rearrangement versus Carbene Formation in Gold-Catalyzed Transformations of Alkynyl Aryl Sulfoxides: Mechanistic Studies and Expanded Reaction Scope. *J. Am. Chem. Soc.* **2013**, *135*, 8512-8524.

2. Hohenberg, P. and Kohn, W. Inhomogeneous Electron Gas. *Phys. Rev.* **1964**, *136*, B864-B871.

3. Tran, H.; Toland, A.; Stellmach, K.; Paul, M. K.; Gutekunst, W.; Ramprasad, R. Toward Recyclable Polymers: Ring-Opening Polymerization Enthalpy from First-Principles. *J. Phys. Chem. Lett.* **2022**, *13* (21), 4778-4785.

4. Stellmach, K. A.; Paul, M. K.; Xu, M.; Su, Y-L.; Fu, L.; Toland, A. R.; Tran, H.; Chen, L.; Ramprasad R.; Gutekunst, W. R. Modulating polymerization thermodynamics of thiolactones through substituent and heteroatom incorporation. *ACS Macro Letters*, **2022**, *11*, 895-901.

5. Sahu, H.; Shen, K. H.; Montoya, J. H.; Tran, H., and Ramprasad, R. Polymer structure predictor (psp): a python toolkit for predicting atomic-level structural models for a range of polymer geometries. *J. Chem. Theor. Comput.* ***2022***, *18* (4), 2737-2748.

6. Kresse, G. and Furthmüller, J. Efficiency of ab-initio total energy calculations for metals and semiconductors using a plane-wave basis set. *Comput. Mater. Sci.* **1996**. *6*, 15-50.
